# A scoping review of adoption of climate-resilient crops by small-scale producers in low- and middle-income countries

**DOI:** 10.1038/s41477-020-00783-z

**Published:** 2020-10-12

**Authors:** Maricelis Acevedo, Kevin Pixley, Nkulumo Zinyengere, Sisi Meng, Hale Tufan, Karen Cichy, Livia Bizikova, Krista Isaacs, Kate Ghezzi-Kopel, Jaron Porciello

**Affiliations:** 1 Cornell University, Ithaca, NY USA; 2 CIMMYT, Mexico City, Mexico; 3 World Bank, Washington, DC USA; 4 University of Notre Dame, Notre Dame, IN USA; 5 USDA-ARS, East Lansing, MI USA; 6 International Institute for Sustainable Development, Winnipeg, Manitoba Canada; 7 Michigan State University, East Lansing, MI USA

**Keywords:** Agriculture, Plant sciences, Plant breeding

## Abstract

Climate-resilient crops and crop varieties have been recommended as a way for farmers to cope with or adapt to climate change, but despite the apparent benefits, rates of adoption by smallholder farmers are highly variable. Here we present a scoping review, using PRISMA-P (Preferred Reporting Items for Systematic review and Meta-Analysis Protocols), examining the conditions that have led to the adoption of climate-resilient crops over the past 30 years in lower- and middle-income countries. The descriptive analysis performed on 202 papers shows that small-scale producers adopted climate-resilient crops and varieties to cope with abiotic stresses such as drought, heat, flooding and salinity. The most prevalent trait in our dataset was drought tolerance, followed by water-use efficiency. Our analysis found that the most important determinants of adoption of climate-resilient crops were the availability and effectiveness of extension services and outreach, followed by education levels of heads of households, farmers’ access to inputs—especially seeds and fertilizers—and socio-economic status of farming families. About 53% of studies reported that social differences such as sex, age, marital status and ethnicity affected the adoption of varieties or crops as climate change-adaptation strategies. On the basis of the collected evidence, this study presents a series of pathways and interventions that could contribute to higher adoption rates of climate-resilient crops and reduce dis-adoption.

## Abstract (web summary)

Climate-resilient crops are essential for farmers to adapt to climate change. This scoping review identifies extension services and outreach as the most important factors for their adoption by small-scale producers in low- and middle-income countries.

## Main

Agriculture and food production are highly vulnerable to climate change. Extreme weather events such as droughts, heat waves and flooding have far-reaching implications for food security and poverty reduction, especially in rural communities with high populations of small-scale producers who are highly dependent on rain-fed agriculture for their livelihoods and food. Climate change is expected to reduce yields of staple crops by up to 30% due to lower productivity and crop failure^1^. Moreover, the projected global population growth and changes in diets toward higher demand for meat and dairy products in developing economies will stretch natural resources even further, increasing demands on food production and food insecurity^2^. To cope with climate change, farmers need to modify production and farm management practices, such as adjusting planting time, supplementing irrigation (when possible), intercropping, adopting conservation agriculture, accessing short- and long-term crop and seed storage infrastructure, and changing crops or planting more climate-resilient crop varieties.

This scoping review examines the conditions that have led to the adoption of climate-resilient crops over the past 30 yr in lower- and middle-income countries. For all countries, but especially those that rely on domestic agriculture production for food security, one of the most critical and proactive measures that can be taken to cope with food insecurity caused by unpredictable weather patterns is for farmers to adopt climate-resilient crops. Climate-resilient crops and crop varieties have enhanced tolerance to biotic and abiotic stresses^3^ (Box 1). They are intended to maintain or increase crop yields under stress conditions and thereby provide a means of adapting to diminishing crop yields in the face of droughts, higher average temperatures and other climatic conditions^4^. Adoption of climate-resilient crops, such as early-maturing cereal crop varieties, heat-tolerant varieties, drought-tolerant legumes or tuber crops, crops or varieties with enhanced salinity tolerance, or rice with submergence tolerance, can help farmers to better cope with climate shocks. Climate-resilient crops and crop varieties increase farmers’ resilience to climate change, but despite their benefits, adoption rates by small-scale producers are not as high as expected in some cropping systems^4–6^. In this study, we focus on scoping (reviewing and synthesizing) the published evidence on the adoption of climate-resilient crops and crop varieties from climate-vulnerable countries and countries that have experienced climate-related impacts as determined by 45 indicators established by the Notre Dame Global Adaptation Initiative.

Overall, we find that the most important determinants of adoption of climate-resilient crops are the availability and effectiveness of extension services and outreach, education level of heads of households, including some awareness of climate change and adaptation measures, and farmers’ access to inputs, especially seeds and fertilizers. On the basis of the collected evidence, this scoping review presents a series of pathways and interventions that can contribute to higher adoption rates of climate-resilient crops and reduce dis-adoption (Box 2).

Box 1 Definitions and assumptions

- **Small-scale food producers.** Definitions of small-scale food producers in the literature are mostly based on four criteria: land size, labour input (especially of family members), market orientation and economic size^2^. Land size is the most commonly used criterion. The clear majority of definitions of small-scale food producers are based on the acreage of the farm and/or a headcount of the livestock raised. Sometimes an arbitrary size is created (commonly 2 hectares or less), but otherwise a relative measure is used, which considers the average size of landholdings in the country, as well as a poverty measure (farms that generate 40% or less of the median income). A second important criterion of small-scale producer is the source of the labour used on the farm (whether it is provided by the household that runs the farm or workers who are paid a wage). A third criterion is the extent to which the farm output is sold to market rather than consumed by the farm household or bartered with neighbours (some authors caution that this is also contextual and many small-scale producers are engaged in commercial markets). A fourth criterion is economic size (the value of the farm’s production)^56^.
- **Climate-vulnerable countries** are countries that are considered to be vulnerable to climate change. The ND-GAIN index presents a list of countries ranked by vulnerability to climate change and readiness to respond (https://gain.nd.edu/our-work/country-index/rankings/).
- **Climate resiliency** is the capacity for a socio-ecological system to absorb stresses and maintain function in the face of external stresses imposed on it by climate change, and adapt, reorganize and evolve into more desirable configurations that improve the sustainability of the system, leaving it better prepared for future climate change impacts.
- **Climate change adaptation** includes planned or autonomous actions that seek to lower the risks posed by climatic changes, either by reducing exposure and sensitivity to climate hazards or by reducing vulnerabilities and enhancing capacities to respond to them. Adaptation also includes exploiting any beneficial opportunities presented by changing climates.
- **Climate-resilient crops** are crops and crop varieties that have enhanced tolerance to biotic and abiotic stresses. They are intended to maintain or increase crop yields under stress conditions such as drought, flooding (submergence), heat, chilling, freezing and salinity, and thereby provide a means of adapting to diminishing crop yields in the face of droughts, higher and lower than seasonal temperatures, and other climatic conditions^3,57^.
- **Climate-smart agriculture** is an approach or set of practices aimed at increasing agricultural productivity and incomes sustainably, while building resilience and adapting to climate change conditions and reducing and/or removing greenhouse gas emissions where possible^6^.
- **Conservation agriculture** is a farming system that promotes minimum soil disturbance (that is, no tillage), maintenance of a permanent soil cover, and diversification of plant species; for instance, through crop rotation^58^.
- **Adoption** is the stage at which technology has been selected and is being used over a sustained period by an individual or an organization. Adoption is more than acceptance; it is inclusion of a product or innovation among the common practices of the adopter.
- **Gender** refers to the social relations between men and women, boys and girls, and how this is socially constructed. Gender roles are dynamic and change over time.
- **Agricultural extension** is a form of outreach that shares research-based knowledge with farmers and communities in order to improve agricultural practices and productivity. The approach to delivering these services varies in terms of farmer participation and engagement. This range includes technology transfer, advisory, experiential and iterative learning, farmer-led extension services (such as farmer field schools), and facilitation, in which farmers define their own problems and develop their own solutions.

Box 2 Summary methods

- A double-blind title and abstract screening was performed on 5,650 articles that were identified through a comprehensive search of multiple databases and grey literature sources and then uploaded to the systematic review software Covidence. The full search protocol is described in the Supplementary Information.
- The resulting 886 articles were subjected to a second round of full-text screening, and 684 articles that did not meet the inclusion criteria were excluded, leaving 202 articles that were read in full and included in the qualitative synthesis.
- We performed data extraction on each of the 202 included studies. A data-extraction template (available in the Supplementary Information) was developed to document the data, study type and context of each citation and all themes of interest.
- The extracted data were qualitatively summarized on the basis of emerging themes and with the aim of providing recommendations to donors and policy makers.
- Among the 684 articles that were excluded at the full-text screening phase, 230 were excluded because they did not include an explicit analysis of factors for climate-resilient crop adoption and 204 were excluded because there was no explicit focus on crops, varieties, seed, planting materials or germplasm.

The inclusion criteria for this study were:

1. The study focus includes population of small-scale food producers, as defined in the protocol
2. The study was published after 1990 (1990 was the year the Intergovernmental Panel on Climate Change (IPCC) produced its first report on climate change).
3. The study includes original research (qualitative and quantitative reports) and/or a review of existing research, including grey literature.
4. An explicit focus or clear relevance on climate change resilience or climate change adaptation, as defined in the protocol.
5. An explicit focus on crops, varieties, seed, planting materials or germplasm.
6. The study mentions factors for adoption, as defined in the protocol.
7. The area of focus of the study includes target populations in lower- and middle-income countries, as defined by the World Bank.

## Results

A scoping review aims to explore the key concepts underpinning a research area and the main sources and types of evidence available^7^. Established scoping review methods provide an evidence-based framework for systematically searching and thematically characterizing the extent, range and nature of existing evidence. A PRISMA-P protocol for this scoping review^8^ was registered on 4 June 2019 on the Open Science Framework. We performed double-blind title and abstract screening of 5,649 citations, selecting 568 papers for full-text screening using a priori inclusion and exclusion criteria; 202 papers met the inclusion criteria for data extraction. The inclusion and exclusion criteria are available in the protocol (Methods and Supplementary Information), and the data-extraction procedure and the PRISMA flow diagram of included and excluded studies are presented in the Supplementary Information.

Of the 202 papers included, 89% were published in peer-reviewed journals and 11% were published in the grey literature. Eighty-seven studies used mixed methods, 82 used quantitative methods and 33 studies used qualitative methods.

### Evidence of adoption of climate-resilient crops

**Fig. 1:**
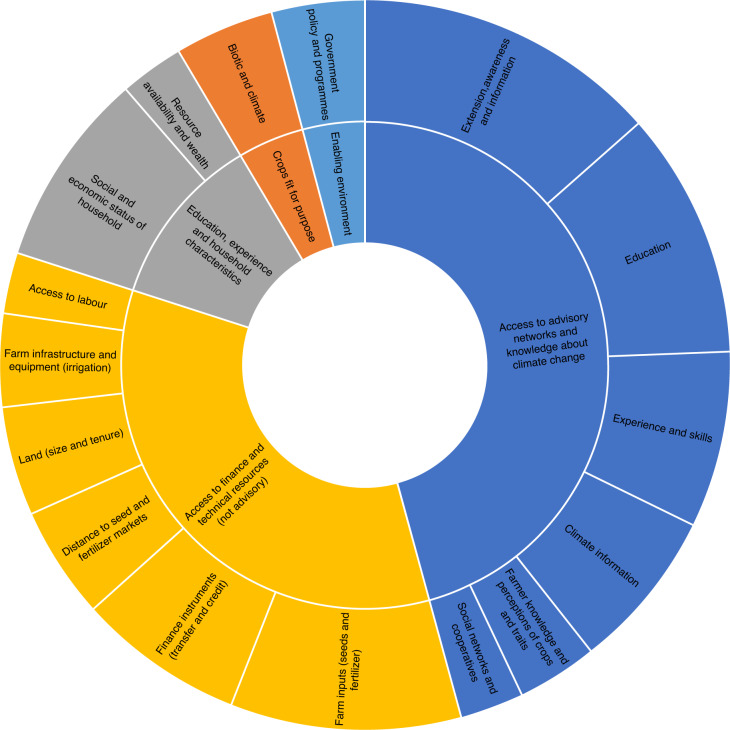
Summary of determinants of adoption of climate-resilient crops and crop varieties by farmers. The inner ring outlines the five broad categories to which the 29 social and economic factors are mapped. The outer ring shows the factors within each broad category that were most frequently mentioned across the included studies. The relative area occupied by categories indicates their relevance. Charts with the full data and frequencies for each category are presented in the Supplementary Information. For illustrative purposes, factors mentioned in less than 20% of studies as determinants of adoption were excluded from this figure.

**Extended Data Fig. 1:**
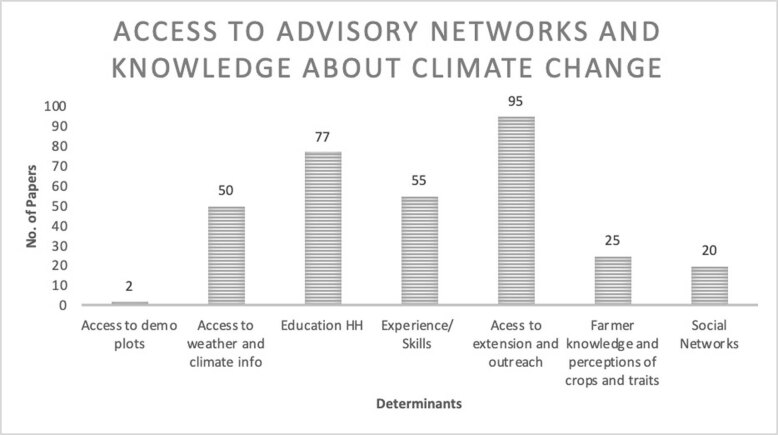
Access to advisory networks and knowledge about climate change. Social determinants captured in this graph are a small-scale producers access to demonstration plots, access to weather and climate info, education of the head of household or respondent if not head of household, experience and skills of head of household or respondent, access to extension and outreach, access to social networks including co-operatives, and a knowledge and perceptions of crops and traits.

**Extended Data Fig. 2:**
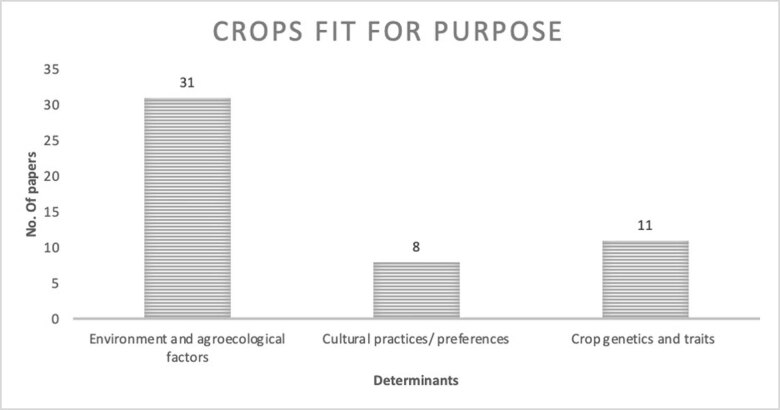
Crops fit for purpose. Social determinants captured in this graph include farmer’s selection of a CR crop or variety based on environmental and agro-ecological conditions, cultural practices and preferences about CR crops and varieties, and selection based on knowledge about a crop traits.

**Extended Data Fig. 3:**
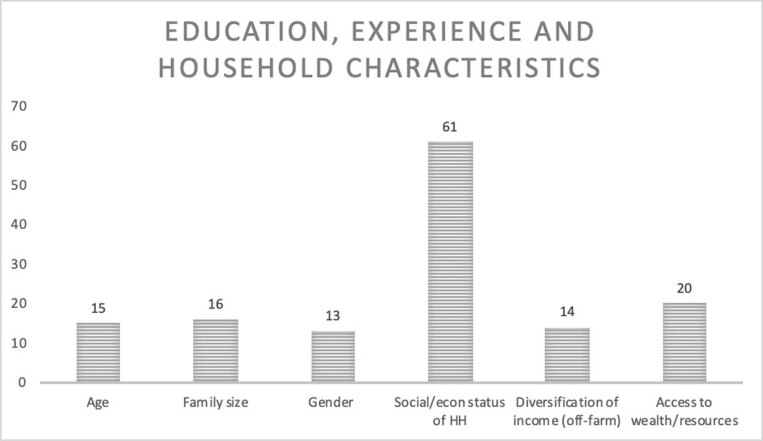
Education, experience and household characteristics. The social determinants captured in this graph include age of head of household or respondent, family size, gender, social and economic status of household, and diversification of household income.

**Extended Data Fig. 4:**
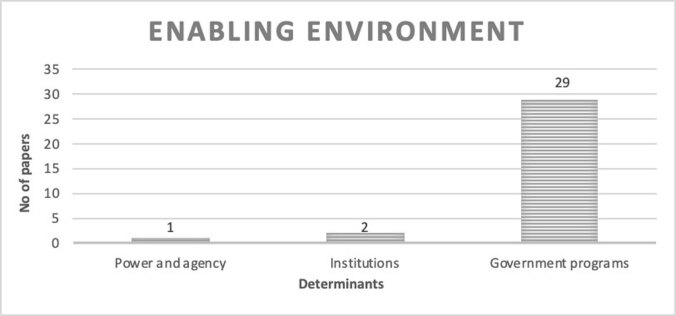
Enabling environment. The determinants captured in this graph include a farmer’s reported power and agency, access to institutions, and access to government programs.

**Extended Data Fig. 5:**
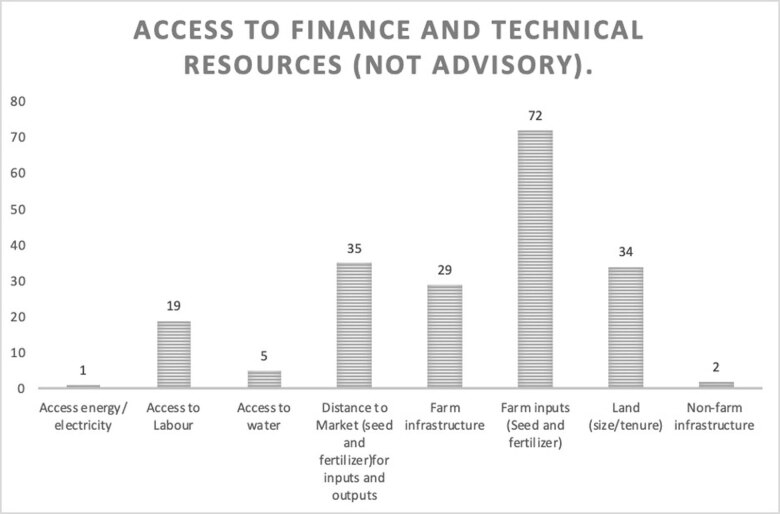
Access to finance and technical resources (not advisory). The determinants in this chart include access to energy and electricity, access to labour, access to water, distance to market for inputs and outputs, farm infrastructure, farm inputs (seeds and fertilizer), land (size and tenure), non-farm infrastructure, access to finance (transfers and credit).

Of the 29 evaluated potential social and economic factors related to adoption, interventions related to the availability, effectiveness and access to agricultural extension services were the most prominent determinants of the adoption of climate-resilient crops in low- and middle-income countries. Nearly 50% of the studies identified extension services and awareness outreach as important factors for the effective adoption of climate-resilient crops in low- and middle-income countries (Fig. 1). The individual figures per characteristic are presented in detailed summary graphs in Extended Data Figs. 1–5. The determinants are plotted in bar charts to provide additional context and visualization. The unit of analysis is per study, and a single study can report on multiple determinants.

**Fig. 2:**
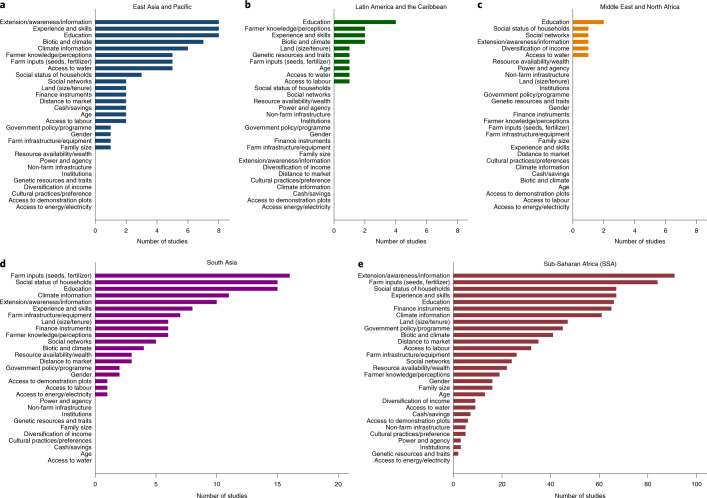
Relevance of social, environmental and economic determinants of adoption of climate-resilient crops by region. **a**–**e**, Individual determinants are ranked from highest to lowest number of studies in the regions: East Asia and Pacific (**a**), Latin America and the Caribbean (**b**), Middle East and North Africa (**c**), South Asia (**d**) and sub-Saharan Africa (**e**).

The principal factors determining adoption of climate-resilient crops or crop varieties were largely consistent across the three regions with robust numbers of publications: sub-Saharan Africa, South Asia and East Asia. The most important determinants across these regions were, in order of importance: (1) access to extension services or information about options, (2) education level of head of household, (3) access to needed farm inputs, (4) experience and skills of farmer, (5) social status, and (6) access to climate information (Fig. 2). Access to extension services and information about options, and education level of head of household were among the top five determinants for adoption for all three regions. Access to farm inputs was the first and second most important determinants for adoption in South Asia and sub-Saharan Africa, respectively, but was only sixth most important for East Asia. Experience and skills of farmers were first and third most important determinants for adoption in East Asia and sub-Saharan Africa, respectively, and sixth most important in South Asia. Social status was highly important in South Asia and sub-Saharan Africa, but only moderately important for determining adoption of technologies in East Asia. Although there were few papers and thus limited information for Latin America and Middle East and North Africa regions, the education level of the head of household was cited as the most important determinant for adoption in both regions.

**Fig. 3:**
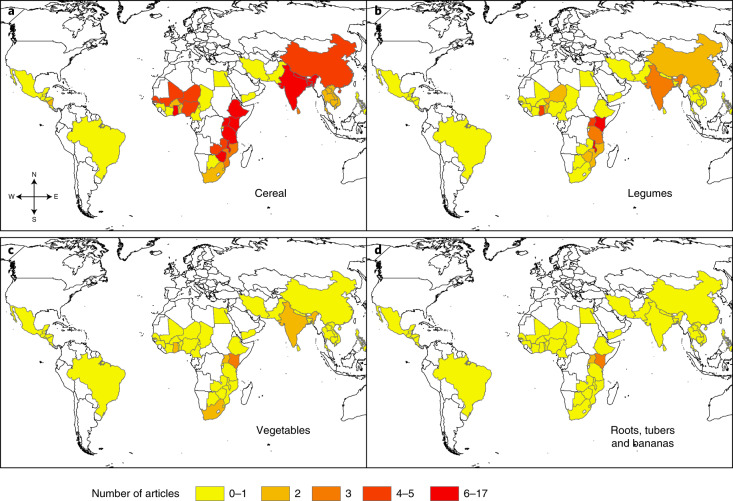
Map of evidence distribution by country and crops. **a**–**d**, Countries are colour-coded from yellow to red based on number of relevant studies involving cereal (**a**), legumes (**b**) vegetables (**c**) and roots, tubers and bananas (**d**).

The climate-resilient crops are included in this scoping review on the basis of data found in the included papers (Fig. 3). We classified them as cereals (maize, rice, grain (general), wheat, millet, sorghum barley and teff), legumes (soybean, chickpeas, cowpea, common beans, mung beans and groundnut), vegetables and fruits (tomato, eggplant, pepper, cocoa, mango, clover, garlic, mustard, pea, onion, saffron, green grams and cola nut) and roots, tubers and bananas (banana, plantain, yam, sweet potato, cassava and potato). Thirty-three per cent of the studies did not report on a specific crop or variety in their research; of the studies that did report on a specific crop or variety, 67% reported on cereals only. Despite their importance for food security and nutrition, less than 1% of the studies reported on legumes only and 25% reported on a combination of cereals and legumes, roots, tubers, bananas, vegetables and fruits. We also assessed the 202 papers to determine the purpose of the crops as primarily for human consumption (44%), for human consumption and animal feed (26%) or not clearly stated (30%).

**Fig. 4:**
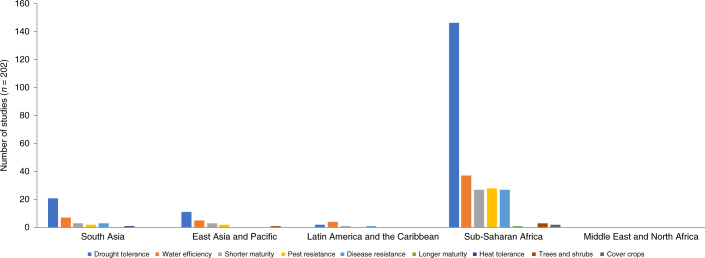
Climate-resilient trait or crop change adopted in response to climate change. Studies are divided into the same geographical regions as in Fig. 2.

Climate-resilient crops and crop varieties were adopted to cope with abiotic stresses such as drought, heat, flooding, salinity and shorter growing season (early-maturing crops), as well as pests associated with changes in weather or climate patterns (disease and pest resistance) (Fig. 4). Climate-resilient crops and crop varieties were also adopted to address general challenges associated with climate change and crop system sustainability, such as to improve moisture retention in soil, improve soil quality, and reduce erosion (planting of cover crops and legumes and to reduce vulnerability to food insecurity). The most studied trait in the dataset was drought tolerance, followed by water-use efficiency and earlier maturity. Adoption of early-maturing crops enables farmers to cope with climate change-induced weather variability by allowing them to adjust planting dates when rains are delayed and reducing the chances of yield losses caused by drought or heat waves late in the growing season. Changing of planting dates was identified in 32% of the papers as a strategy to cope with climate change.

In general, the evidence suggests that farmers do not adopt a new crop or crop variety without changing other practices. A total of 136 papers (67%) describe that farmers adopt climate-resilient crops in conjunction with other climate-resilient technologies such as climate-smart agriculture (CSA) schemes and conservation agriculture (CA). Other climate-resilient technologies included: planting of trees and shrubs, reduced or increased investment in livestock and modified planting dates and irrigation (Table 1).

**Table 1:** Adoption of climate-resilient crops as part of broader climate-resilience strategies

Type of response to climate change	Percentage of papers that list the response^a^	Examples of specific activities associated with each response to climate change
New variety planted	24%	Introduction of a new variety of an existing crop to the farmer
Modified planting activities	32%	Change in planting date, crop diversification, crop rotation and intercropping
Irrigation and water management	32%	Water conservation strategies, irrigation, micro-irrigation, water harvesting and improving drainage
Seeking off-farm work or migration	5%	Outmigration, seeking off-farm employment and diversification of activities beyond the farm
Storage and infrastructure development	5%	Crop storage development and improvement, community sharing and road building
Use of fertilizers and pesticides	16%	Use of fertilizers, including manure and pesticides, and change in use of fertilizers, compost manure and green manure
Planting trees	12%	Planting shade trees and agroforestry

^a^Most papers listed multiple types of response to climate change; thus, the total is above 100%.

### Seed and adoption of climate-resilient crops

Seventy-three papers mentioned the topic of seed. The major themes associated with seed that emerged with direct evidence drawn from the papers are summarized in Table 2. Access to and availability of seed were the most prevalent themes, with 60% of papers mentioning these as issues in the adoption of climate-resilient strategies. Social networks such as farmers’ organizations or co-operatives, as well as access to information, were also reported as facilitators of adoption. These themes refer to different social groups and ways in which farmers can exchange seed or get information about seed.

**Table 2:** Seed factors associated with adoption of climate-resilient crops and crop varieties

Emergent themes about seed	Summary of the evidence
Access	Access to seed or the ability to afford seed was a principal barrier for small-scale farmers’ adoption of climate-resilient varieties. Several papers mentioned that cost was even more challenging for women and farmers with fewer assets, smaller parcels of land or lower economic status. At least four papers suggested seed subsidies as a strategy to improve access to seed^35–38^.
Availability	Availability, or the ability to acquire seed on time, in the quantity needed and within reasonable proximity, was a determinant of adoption related to seed. Community seed banks also enhanced availability of seed.
Social networks	Participation in social networks that enable the exchange of seed was a climate-resilient strategy for farmers. Participation in social networks, which included community-based seed banks, seed organizations, farmer groups and intra-village or neighbour networks improved the adoption of seed (or new varieties for climate resilience), and these social networks also increased the spread of seed that was distributed as part of development projects. Conversely, one paper reported that seed did not spread beyond the immediate beneficiaries of the project^39^. Another report stressed the importance of reciprocity within strong social networks as important for maintaining access to seed^40^, and several others recommended supporting social networks to strengthen seed systems^40–44^. According to three papers, community seed banks strengthened social networks for exchange, provided landraces for participatory crop improvement, and increased the availability of seed^44–46^. Integration of informal and formal seed system elements is important because most of the seeds planted by small farmers are uncertified and sourced through informal seed system channels or social networks^47^. Social networks also have an important role in enhancing farmers’ access to information.
Information	Farmers lacked information about varieties, adaptation and attributes, or did not know where to acquire seed. Extension services, seed companies, seed suppliers and seed traders were a source of information about seed, and in some cases increased use of seed and other management practices. In a few cases, there was evidence that access to extension services positively influenced the use of certified seed, and in another, the authors suggested that extension services could help farmers become aware of different adaptive strategies and help in the distribution of seed of improved varieties.
Gender	Few papers explicitly linked gender and seed. Improved seed was more difficult to acquire for female-headed households and women were less likely to use improved seed or have access to extension services; small, affordable seed packs were suggested as a potential solution.
Strategy	Improved or hybrid seed and exchanging seed with other villages were considered to be climate-resilience strategies for farmers.
Policy	A few papers discussed agricultural policies related to seed, arguing that policies should enable the seed sector to provide suitable varieties and aim to increase the availability of funds for seed distribution research and access to improved seed, and one paper indicated that government policies restrict farmers options for obtaining their preferred seed^48–50^.
Experience	One paper indicated that farmers’ experience had a positive effect on adoption of new seed, whereas another indicated the opposite^9,51^.
Seed or variety attributes	Four papers reported on concerns related to the attributes of the hybrid seed varieties and their adaptation to the environment, suitability for storage, flour to grain ratio, and other processing issues^52–54^. One study found that farmers favour composite varieties and local landraces under conditions of abiotic stress^55^.
Seed sovereignty	One paper discussed issues related to seed sovereignty, reporting that farmers wanted a say in where seed comes from and were resistant to the use of transgenic crops. They expressed a belief that seed industries are appropriating a resource that belongs to humanity. Autonomy is highly valued by these communities, and local varieties are valued in part for their contribution to maintaining independence from commercial hybrid seed sources^40^.

### Social differences and adoption of climate-resilient crops

About 53% of studies reported that social differences (such as sex, education and age of household head) influence adoption of varieties or crops as mitigation strategies against the effects of climate change, whereas 30% of studies did not report any effect of social difference. Fifteen per cent of studies did not include data on social differences. Of the studies that identified social differences as influencing adoption of climate-resilient crops and crop varieties, education (22%), sex (28 %), age (24%) and family size (14%) emerged as the most important factors. Income (6%), access to information (5%), marital status (2%) and experience (2%) were also mentioned, but much less frequently. We examined the papers for sex disaggregation of data, in which sex of household heads was considered. Forty-five per cent of studies reported on the sex of respondents, with 39% reporting on both male and female household heads, 5% including men only, and only 1% of studies including only female respondents. Most of the studies explored social differences only superficially, by including variables in surveys, but few substantiated these findings with follow-up qualitative research to understand the social dynamics driving the observed adoption decisions.

The studies largely concur that socio-economic status of farmers plays a large part in their adoption of climate-resilient technologies. Thirty-one per cent of the studies highlighted the socio-economic status of farmers. Various studies indicated that a nuanced understanding of the socio-economic status of farmers is vital for the targeting of climate-resilient crop technology interventions and their adoption and sustainability in practice. Thirteen studies reported a positive effect of farmer income on adoption. Farmers with access to finance, such as risk transfers (for example, insurance or remittances) and credit (for example, bank loans or community loans), were more likely to adopt climate-resilient crop technologies. Farmers who reported constrained credit were less likely to grow modern crops and more likely to cultivate local varieties^9^. This is partly because the lack of cash or credit may prevent farmers from using purchased inputs^10^.

### Evidence on the dis-adoption of climate-resilient crops

Dis-adoption of climate-resilient crops and crop varieties was discussed in 12 of the 202 papers included in our evidence synthesis. The major reasons for dis-adoption included technology not meeting expectations due to poor performance or quality of the technology or variety (8 papers), government policies (3 papers), technical constraints (2 papers), labour shortages (1 paper) or financial constraints (1 paper). Eight of the twelve studies indicated that dis-adoption was specifically due to the performance of a crop variety, and four of these eight studies indicated that the varieties’ performance under stress conditions did not meet farmers’ expectations^10–13^.

## Discussion

The primary goal of this scoping review was to identify factors in adoption of climate-resilient crops in climate-vulnerable countries. Insights into these factors may inform the design of interventions aimed at equipping farmers to adopt climate-resilient technologies before experiencing devastating impacts of climate change and encourage adoption best practices^14,15^.

We show that there is a predominance of cereals in reported studies on adoption of climate-resilient crops (67%). Only 1% of the studies report on legumes only; otherwise, they are considered only in combination with other crops. This may reflect the dominance of cereals in staple foods across the world and biases towards the study of such crops and in the development of improved climate-resilient crop varieties. However, this is a concerning trend given that some legumes, roots and tuber crops (for example, cassava, bambara groundnuts and beans) that are largely neglected in the studies have known climate resilience, are sources of high-quality nutrition and provide more well-established environmental benefits than cereals, such as soil enrichment.

About 50% of the studies included in this scoping review identified agricultural extension and awareness outreach as the most relevant factor for adoption of climate-resilient technologies in low- and middle-income countries. Agricultural extension links farmers with the latest research and engages in a translational practice to make complex information more accessible to farmers. It has been shown that farmers who have access to early-warning systems such as weather forecast systems can better cope and adapt to a changing climate^16^. Farmers plan better for farming activities, including choice of crop varieties to plant, after having had access to weather forecast information (for example, from a community-managed weather station). Emerging digital technologies provide an opportunity to use information and communications technology-enhanced extension and climate services that can provide timely information that farmers can use for decision making and to adapt their farming practices. These could also improve efficiencies of extension services while also reducing their cost. Poor funding for extension services in the developing world have limited farmers’ access to training and expert guidance on emerging technologies^17^. Partnerships with other emerging players in information exchange, such as telecommunications companies and non-governmental organizations, will be key.

Farmers generally tend to be risk averse, which leads to limited investment and adoption of improved agricultural production technology^18^. Experienced farmers use precautionary strategies to protect against the possibility of catastrophic loss in the event of a climatic shock and thus optimize management for average or likely conditions, but not for unfavourable conditions. These ex ante, precautionary strategies include selection of crops and cultivars and improved production technology^18^^.^

In general, there is widespread agreement that aside from the useful experience that farmers gain from the time they have spent in farming, their experience with climatic shocks is key to their adoption of climate-resilient technologies. Many studies showed that farming experience is influential in adoption and utilization, and previous experiences with environmental shocks such as drought can influence adoption of climate-resilient crops and crop varieties. The more experience farmers have with climatic shocks, the more likely they are to be receptive to the adoption of related climate-resilient technologies. For example, experience with drought shock in the agro-ecological zone of Brong Ahafo, Ghana, increased the probability of adoption of drought-tolerant varieties by 15%, and farmers reported that drought shock was the primary reason for adoption of drought-tolerant varieties^19^.

It has been widely acknowledged that education levels of farmers have a positive correlation with technology adoption, and our synthesis demonstrates that this is also relevant for the adoption of climate-resilient crops^16,20–22^. Highly educated heads of households are more likely to readily accept and access information about new technologies in a shorter period of time than less educated heads of households; education was measured as educational attainment and reported in 49% of the studies. A study based in Zimbabwe showed a 52% decrease in production of traditional sorghum varieties in favour of new varieties better suited to drier conditions for every additional year of schooling, and a 5% increase in growing new early-maturing varieties^23^.

Changing crop varieties is one of the most frequently cited climate-resiliency strategies for both men and women farmers, but women are more likely to adopt such strategies when they are aware of climate-adaptation options^24^. Other intersectional variables such as marital status, education and age, in combination with gender, influenced whether improved seed was grown by households^25^. A major shortcoming of the reviewed literature is that most studies included women only when they were household heads. Definitions of household headship are variable, and when women are only included as household heads, their views do not necessarily represent the views of women who live in male-headed households^26^. A large majority of women live in male-headed households, and their views are rendered invisible through this practice^27^. For example, young, poor women who were household heads were the least likely to adopt drought-tolerant maize in Uganda, whereas spouses of male household heads influenced adoption decisions on their husbands’ fields^9^. Only a few studies paid attention to intra-household dynamics, gender roles and relations, and how these shape adaptation decisions^9,28^. This limited attention on intra-household gender dynamics and decision making around climate-resilient seed adoption skews the conclusions and recommendations, as the literature does not equally represent the challenges and views of women.

Seed policies in many countries focus on strengthening formal, national seed systems that rely on variety-release mechanisms, seed certification policies and seed companies for distribution. These types of seed systems remain difficult to access for many farmers, and evidence from the papers in this scoping review suggests that strengthening local seed systems is essential. Local seed systems rely on social networks to ensure multiple options to access seed of a range of climate-resilient crops and varieties, including local landraces and improved seed. Thus, context specificity is important for seed systems, as it is for almost all factors influencing adoption of climate-resilient crops and varieties.

The determinants of adoption that we identified are, in many cases, context-specific and therefore implementation of specific interventions is most successful when they are tailored to their environment and the cropping system. Seemingly contradictory or opposing (positive and negative) effects of each determinant of adoption were commonly reported among—and sometimes within—studies. Sex, age, education, years of farming experience and indicators of socio-economic status or wealth (assets) all affected decisions to adopt climate-resilient technologies in context-specific and sometimes opposite ways, depending on interacting environmental, policy and household factors. For example, equal and sizable numbers of studies (13 each) identify positive and negative effects of age on adoption. Whereas some studies identified older farmers to be more reluctant to adopt new technologies, other studies found that the earned experience, broad social networks and accumulation of wealth associated with older farmers may explain a positive effect on adoption. Extension and access to information about climate-resilient technologies and weather might be exceptions to this trend, as these determinants seem to transcend context-specific implementation. The resulting conclusion is that there is no ‘one size fits all’ recommendation to ensure adoption of climate-resilient crops and crop varieties, and interventions are unlikely to uniformly benefit all climate-vulnerable farmers (Table 3). This is consistent with the large number of papers in this study that reported farmers adopting climate-resilient crops as part of broader climate-resilient strategies.

**Table 3:** What does the evidence say? Specific undertakings to improve adoption of climate-resilient crops and crop varieties

Types of suggested specific actions to increase adoption of climate-resilient crops	Number of papers (%)^a^
Providing extension programmes to support the uptake of climate-resilient crops	38 (15.8%)
Providing access to financial instruments (credit, insurance and loans)	29 (12.1%)
Implementing community programmes to support the uptake of climate-resilient crops	28 (11.7%)
Promoting of germplasm conservation programme and research	25 (10.4%)
Providing access to fertilizer, pesticides and other inputs	20 (8.3%)
Awareness raising about climate change, weather and impacts	19 (7.9%)
Awareness raising of climate-resilient crops	15 (6.3%)
Promoting infrastructure development, especially irrigation and roads	14 (5.8%)
Targeted programmes on youth and women to engage them in climate-resilient crops	14 (5.8%)
Providing access to climate-resilient seed	13 (5.4%)
Providing low-cost climate-resilient options for farmers	13 (5.4%)
Livestock-focused initiatives to address fodder development in the context of climate change	6 (2.5%)
Linking support for climate-resilient crops as part of poverty-reduction efforts	6 (2.5%)

^a^Multiple potential activities were occasionally listed together.

Climate resiliency at farm level is essential to achieve food security and improve livelihoods of rural communities, especially in countries and communities that depend on local agricultural production to ensure household income and achieve daily adequate caloric intake and balanced nutrition. Understanding the factors contributing to adoption and dis-adoption of climate-resilient crops provides opportunities to increase adoption and reduce the impact of climate change on rural communities in developing countries. The most important determinants of adoption of climate-resilient crops based on our analysis are the availability and effectiveness of extension services and outreach, followed by education levels of heads of households, farmers’ access to inputs, especially seeds and fertilizers, and socio-economic status of farming families. Building resilience to climate change requires a cropping-systems, and more often a farming-systems approach. The results from this scoping review show that the adoption of climate-resilient crops and varieties, in most cases, happens as part of whole-farm and climate-smart agriculture strategies to cope with changing climate. Farmers adopting multiple complementary strategies under climate-smart agriculture help to build highly resilient and sustainable agriculture systems that can respond to shocks associated with climate change and other agricultural challenges^29–31^. Single component intervention programmes or projects are therefore less likely to realize widespread adoption and improvement of resource-poor farmers’ resilience to climate change compared with more holistic, multifaceted approaches that take into consideration the physical, human and socio-economic circumstances of the targeted farmer or farming community. Specific policy recommendations are presented in Box 3.

Box 3 Recommendations

- Access and availability of climate-resilient crops seeds must be combined with relevant and timely advisory services, such as early-warning systems for weather.
- Ensuring that farmers have multiple options to access seeds for a range of climate-resilient crops and varieties is essential. This can be achieved by empowering existing social networks, such as farmer organizations.
- There is no single profile that applies to all farmers. Therefore, extension services will need to continue to evolve to be (1) participatory, (2) information and communications technology enhanced, and (3) partnerships based. This partnership should include various actors, such as women’s groups, universities, the private sector and non-governmental organizations in order to provide customized and appropriate information for diverse needs.
- High-quality studies are needed on how members of households—and not just heads of households—make decisions about how to respond to climate change. This research will fill in the evidence gaps on gender and social differences and reasons for dis-adoption of climate-resilient crops and related technologies, and promote a more diverse group of climate-resilient crops that also provide food security and nutrition, such as legumes and root crops.
- National policies need to support farmers’ access to other assets and services, such as education, land, finance services and diverse income-earning opportunities. Without these provisions, especially education, the adoption of climate-resilient crops and technologies will be limited.
- A multiple-interventions approach is needed if countries want to promote adoption of climate-resilient crops. Farmers do not adopt climate-resilient crop or crop varieties without changing other practices, such as planting dates, water-conserving technologies, planting trees and shrubs, or increasing or decreasing livestock.
- Farmers will not adopt climate-resilient crops solely on the basis of environmental-adaptation qualities. Development and breeding programmes must consider farmer and market trait preferences.
- Mandating disaggregated data collection to identify strategies that are working and who they are working for in agricultural surveys and research will enable policy makers and donors to respond with more appropriate and informed interventions.

## Methods

Unlike a typical narrative review, a scoping review strives to capture all the literature on a given topic and reduce authorial bias. Scoping reviews offer a unique opportunity to explore the evidence in agricultural fields to address questions relating to what is known about a topic, what can be synthesized from existing studies to develop policy or practice recommendations, and what aspects of a topic have yet to be addressed by researchers.

### Evidence synthesis methodology and protocol pre-registration

This scoping review was prepared following guidelines from the PRISMA extension for scoping reviews (PRISMA-ScR)^32^. This framework comprises five steps: identifying the research question; identifying relevant studies; study selection; extracting and charting the data; and collating, summarizing, and reporting the results^33^. The protocol for this scoping review was registered on the Open Science Framework before study selection^8^. The full protocol is available in the Supplementary Information.

### Research question

The guiding question for this scoping review was, ‘what are determinants that lead small-scale producers in low-and middle-income countries to adopt climate-resilient crops and crop varieties?’.

### Information sources, search methods and citation management

An exhaustive search strategy was developed to identify all available research pertaining to facilitators that lead small-scale producers in low- and middle-income countries to adopt climate-resilient crop varieties. Search terms included variations of the key concepts in the research question: small-scale producers, germplasm and climate resilience. The search algorithms were formatted for compatibility with each database so that they may be reproduced in their entirety, and they can be accessed at https://osf.io/sfzcm/. Searches were performed in the following electronic databases by K.G.K.: CAB Abstracts and Global Health (accessed via Web of Science), Web of Science Core Collection (accessed via Web of Science) and Scopus (accessed via Elsevier). A comprehensive search of grey literature sources was also conducted. Search results were de-duplicated to remove redundant citations identified from multiple sources. To facilitate acceleration of the screening process, machine-derived metadata were added to individual citations, for example, identifying populations, geographies, interventions and outcomes of interest. This enabled accelerated identification of potential articles for exclusion at the title- or abstract-screening stage.

### Eligibility criteria and study selection

Studies were included for data extraction and analysis if (1) their focus included a population of small-scale food producers; (2) they were published between 1990 and the start of the search (1990 is when the IPCC first met and produced their first report on climate change); (3) they presented original research (qualitative and quantitative reports) and/or reviewed existing research, including grey literature; (4) they explicitly focused on or were clearly relevant to climate change resiliency or climate change adaptation; (5) they explicitly focused on crops, varieties, seed, planting materials or germplasm; (6) they mentioned factors for adoption; (7) they included target populations in countries classified as lower and middle-income by the World Bank. Studies that did not meet all of the aforementioned inclusion criteria were excluded.

Study selection was performed in two stages. In a first step, articles were uploaded to the systematic review software Covidence, and title and abstract screening was performed by all authors to exclude articles that did not meet all inclusion criteria. Each article was reviewed by two independent authors, and discrepancies were resolved by a third independent author. Full-text screening was then performed by M.A., K.C., S.M., N.Z., H.T., K.P., L.B. and K.I., and inclusion decisions were made by a single reviewer. Studies included in full-text screening were those that met all inclusion criteria or those whose eligibility could not be established during title and abstract screening. The PRIMSA flow diagram in the Supplementary Information presents the study selection process and indicates the number of articles excluded at each phase of screening.

### Data extraction and analysis

A data-extraction template (available in the Supplementary Information) was developed to document the data and study type and context of each citation and all themes of interest. The data extraction first collected data on the paper quality, study location, population socio-economic data of the population and crop and cropping system characteristics. Second, the data-extraction template was used to collect information about the determinants of adoption and associated socio-economic factors influencing the adoption or dis-adoption of the climate-resilient crops. In total, 29 factors and determinants were selected. Additional rater observations and comments were included to increase analysis depth. Finally, raters also recorded policy and programmatic information and recommendations mentioned in the papers to support the adoption of climate-resilient crops. The data-extraction template was tested by the review team before use and data were extracted by the authors. The extracted data were qualitatively summarized on the basis of emerging themes and with the aim of providing recommendations to donors and policy makers. An assessment of study quality is not typically carried out as part of a scoping review^7,34^.

## Supplementary information

Supplementary Information List of included studies, scoping review protocol and data-extraction template.

## Figures and Tables

**Extended Data Fig. 6:**
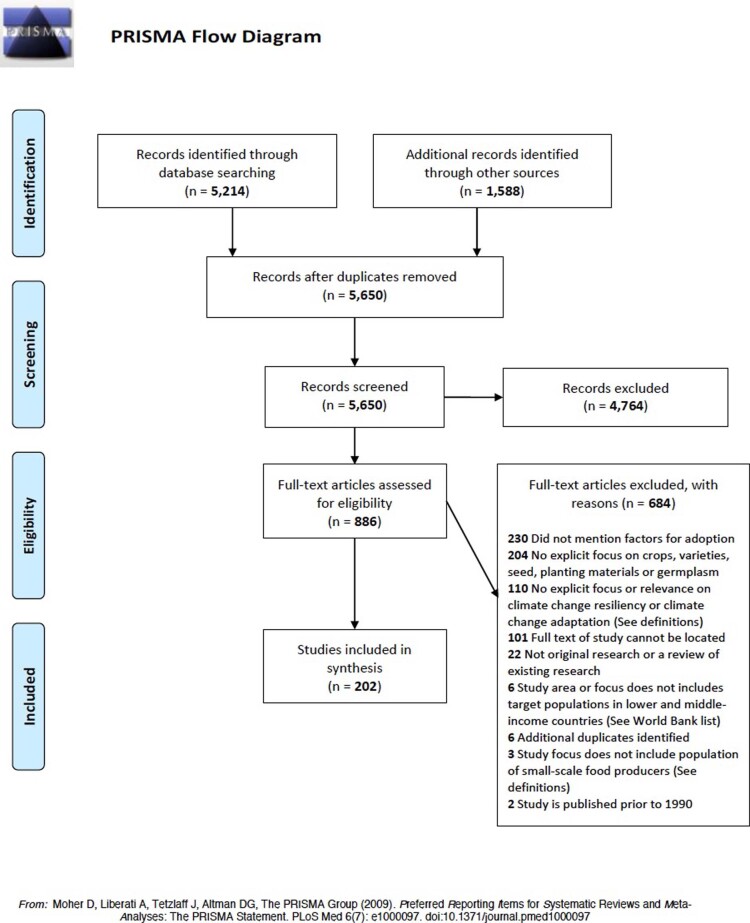
Prisma Flow Diagram.

## Data Availability

The data that support the findings of this study are available from the corresponding author upon request.
