# Supplementary material for: A scoping review of adoption of climate-resilient crops by small-scale producers in low- and middle-income countries
Source: Nat Plants. 2020 Oct 12;6(10):1231–41. doi: 10.1038/s41477-020-00783-z (PMC7553851; doi:10.1038/s41477-020-00783-z)
Supplement: Supplementary file 1 — List of included studies, scoping review protocol and data-extraction template. [file 41477_2020_783_MOESM1_ESM.pdf]

natureresearch

https://doi.org/10.1038/s41477-020-00783-z

Supplementary information

A scoping review of adoption of climate-resilient crops by small-scale producers in low- and middle-income countries

In the format provided by the authors and unedited

List of all included studies

1.Kanwal, V., Pandey, D. & Kumar, S. A Comparative Analysis of Crop Diversification between Flood and Drought Prone Areas of Rajasthan. Indian Journal of Economics and Development 14, 168–174 (2018).

2.Sanchez, A. C., Fandohan, B., Assogbadjo, A. E. & Sinsin, B. A countrywide multi-ethnic assessment of local communities' perception of climate change in Benin (West Africa). Climate and Development 4, 114–128 (2012).

3.Nidhi, S. & Shehrawat, P. S. A study of constraints analysis encountered by gram growers in adoption of improved cultivation practices. International Journal of Agricultural Sciences 14, 394–398 (2018).

4.Abraha, M. T., Shimelis, H., Laing, M. & Assefa, K. Achievements and gaps in tef productivity improvement practices in the marginal areas of Northern Ethiopia: implications for future research directions. International journal of agricultural sustainability 15, 42–53 (2017).

5.Jelliffe, J. L., Bravo-Ureta, B. E. & Deom, C. M. Adaptation and Adoption of Improved Seeds through Extension: Evidence from Farmer-Led Groundnut Multiplication in Uganda. (2015).

6.Tambo, J. A. Adaptation and resilience to climate change and variability in north-east Ghana. International Journal of Disaster Risk Reduction 17, 85–94 (2016).

7.Singh, R. K. et al. Adaptation in rice-wheat based sodic agroecosystems: A case study on climate resilient farmers' practices. (2014).

8.Mugambiwa, S. S. Adaptation measures to sustain indigenous practices and the use of indigenous knowledge systems to adapt to climate change in Mutoko rural district of Zimbabwe. Jàmbá: Journal of Disaster Risk Studies 10, 1–9 (2018).

9.Karanja Ng'ang'a, S., Van Wijk, M. T., Rufino, M. C. & Giller, K. E. Adaptation of agriculture to climate change in semi-arid Borena, Ethiopia. Regional Environmental Change 16, 2317–2330 (2016).

10.Nhamo, N., Donald, M. & Fritz, O. T. Adaptation strategies to climate extremes among smallholder farmers: a case of cropping practices in the Volta Region of Ghana. British Journal of Applied Science & Technology 4, 198–213 (2014).

11.Asfaw, S., McCarty, N., Lipper, L., Arslan, A. & Cattaneo, A. Adaptation to Climate Change and Food Security: Micro-evidence from Malawi. (2013).

12.Wilk, J., Andersson, L. & Warburton, M. Adaptation to climate change and other stressors among commercial and small-scale South African farmers. Regional Environmental Change 13, 273–286 (2013).

13.Menike, L. & Arachchi, K. Adaptation to climate change by smallholder farmers in rural communities: Evidence from Sri Lanka. Procedia food science 6, 288–292 (2016).

14.Komba, C. & Muchapondwa, E. Adaptation to climate change by smallholder farmers in Tanzania. in Agricultural Adaptation to Climate Change in Africa 129–168 (Routledge, 2018).

15.Bryan, E., Deressa, T. T., Gbetibouo, G. A. & Ringler, C. Adaptation to climate change in Ethiopia and South Africa: options and constraints. Environmental science & policy 12, 413–426 (2009).

16.Ochieng, J., Kirimi, L. & Makau, J. Adapting to climate variability and change in rural K enya: f armer perceptions, strategies and climate trends. in Natural resources forum vol. 41 195–208 (Wiley Online Library, 2017).

17.Kassie, B. T. et al. Adapting to climate variability and change: experiences from cereal-based farming in the Central Rift and Kobo Valleys, Ethiopia. Environmental Management 52, 1115–1131 (2013).

18.Ruijs, A., de Bel, M., Kononen, M., Linderhof, V. & Polman, N. Adapting to climate variability: learning from past experience and the role of institutions. (World Bank, 2011).

19.Nyasimi, M. et al. Adoption and dissemination pathways for climate-smart agriculture technologies and practices for climate-resilient livelihoods in Lushoto, Northeast Tanzania. Climate 5, 63 (2017).

20.Raghu, P. T., Erenstein, O., Böber, C. & Krishna, V. V. Adoption and outcomes of hybrid maize in the marginal areas of India. Quarterly Journal of International Agriculture 54, 189–214 (2015).

21.Musa, F. B., Kamoto, J. F., Jumbe, C. B. & Zulu, L. C. Adoption and the Role of Fertilizer Trees and Shrubs as a Climate Smart Agriculture Practice: The Case of Salima District in Malawi. Environments 5, 122 (2018).

22.William, A. et al. ADOPTION OF BAMBARA GROUNDNUT PRODUCTION AND ITS EFFECTS ON FARMERS'WELFARE IN NORTHERN GHANA. (2016).

23.Chazovachii, B., Chigwenya, A. & Mushuku, A. Adoption of climate resilient rural livelihoods through growing of small grains in Munyaradzi communal area, Gutu District. African journal of Agricultural research 7, 1335–1345 (2012).

24.MEENAKSHI, H., SELVARAJ, K. & NAGARAJA, M. Adoption of Drought Coping Mechanisms (Ex-Ante and Ex-Post) and Estimate the Economic Costs of Adoption.

25.Genova, C., Schreinemachers, P. & Afari-Sefa, V. Adoption, yield and profitability of tomato grafting technique in Vietnam. (2015).

26.Esham, M. & Garforth, C. Agricultural adaptation to climate change: insights from a farming community in Sri Lanka. Mitigation and Adaptation Strategies for Global Change 18, 535–549 (2013).

27.Asfaw, S., Di Battista, F. & Lipper, L. Agricultural technology adoption under climate change in the Sahel: Micro-evidence from Niger. Journal of African Economies 25, 637–669 (2016).

28.Kgosikoma, K. R., Lekota, P. C. & Kgosikoma, O. E. Agro-pastoralists' determinants of adaptation to climate change. International Journal of Climate Change Strategies and Management (2018).

29.Basanayak, R. T., Manjunath, L. & Yadav, V. S. An analysis of farmer's opinion and their adaptation behavior to climate change in Bidar district. Agriculture Update 8, 271–273 (2013).

30.Mhike, X., Okori, P., Kassie, G. T., Magorokosho, C. & Chikobvu, S. An appraisal of farmer variety selection in drought prone areas and Its implication to breeding for drought tolerance. Journal of Agricultural Science, Canada 4, 27–43 (2012).

31.Krishna, V. V., Spielman, D. J., Veettil, P. C. & Ghimire, S. An empirical examination of the dynamics of varietal turnover in Indian wheat. (2014).

32.Yuya, B. A. & Daba, N. A. An Evaluation of Climate Mitigation Adoption Technologies in Improving Rural Households' Livelihood Outcomes: The Case of Eastern Oromia, Ethiopia. Turkish Journal of Agriculture-Food Science and Technology 6, 710–718 (2018).

33.Adusei, K. Analyses of perceptions and adaptations to climate change by rice farmers: case study in the Ashanti and Northern Regions of Ghana. (2017).

34.Idrisa, Y. L., Ogunbameru, B. O., Ibrahim, A. A. & Bawa, D. B. Analysis of awareness and adaptation to climate change among farmers in the Sahel Savannah agro-ecological zone of Borno State, Nigeria. (2012).

35.Zizinga, A. et al. Analysis of farmer's choices for climate change adaptation practices in South-Western Uganda, 1980–2009. Climate 5, 89 (2017).

36.Kristjanson, P. et al. Are food insecure smallholder households making changes in their farming practices? Evidence from East Africa. Food Security 4, 381–397 (2012).

37.Mekonnen, D. Assessing local community perceptions on climate change and variability and its effects on crop production in Western Oromia, Ethiopia. in Fifth African Higher Education Week and RUFORUM Biennial Conference 2016," Linking agricultural universities with civil society, the private sector, governments and other stakeholders in support of agricultural development in Africa", Cape Town, South Africa, 17-21 October 2016 1129–1138 (RUFORUM, 2016).

38.McCord, P., Waldman, K., Baldwin, E., Dell'Angelo, J. & Evans, T. Assessing multi-level drivers of adaptation to climate variability and water insecurity in smallholder irrigation systems. World Development 108, 296–308 (2018).

39.Mfitumukiza, D. et al. Assessing the farmer field schools diffusion of knowledge and adaptation to climate change by smallholder farmers in Kiboga District. Uganda. Journal of Agricultural Extension and Rural Development 9, 74–83 (2017).

40.Nyongesa, D., Esilaba, A. O., Emongor, R., Bikketi, E. & Were, K. Assessment of gender and innovations in climatesmart agriculture for food and nutrition security in Kenya: a case of Kalii watershed. International Journal of Agricultural Resources, Governance and Ecology 13, 109–137 (2017).

41.Yapi, A. M., Dehala, G., Ngawar, K. & Issaka, A. Assessment of the economic impact of sorghum variety S 35 in Chad. (1999).

42.Amare, Z. Y., Ayoade, J. O., Adelekan, I. O. & Zeleke, M. T. Barriers to and determinants of the choice of crop management strategies to combat climate change in Dejen District, Nile Basin of Ethiopia. Agriculture & Food Security 7, 37 (2018).

43.Haussmann, B. I. et al. Breeding strategies for adaptation of pearl millet and sorghum to climate variability and change in West Africa. Journal of Agronomy and Crop Science 198, 327–339 (2012).

44.Jiri, O., Mafongoya, P. L. & Chivenge, P. Building climate change resilience through adaptation in smallholder farming systems in semi-arid Zimbabwe. International Journal of Climate Change Strategies and Management (2017).

45.Jayne, T. S., Sitko, N. J., Mason, N. M. & Skole, D. Can Input Subsidy Programs Promote Climate Smart Agriculture in Africa? (2016).

46.Tang, L., Zhou, J. & Yu, X. Can Stress Tolerant Variety Help Reduce Rice Yield Loss from Climate Extremes? Evidence from Chinese Rice Farms. (2016).

47.Naess, L. O., Sullivan, M., Khinmaung, J., Crahay, P. & Otzelberger, A. Changing climates Changing lives: adaptation strategies of pastoral and agro-pastoral communities in Ethiopia and Mali. (2010).

48.Kassem, H. S., Bello, A. R. S., Alotaibi, B. M., Aldosri, F. O. & Straquadine, G. S. Climate change adaptation in the Delta Nile Region of Egypt: Implications for agricultural extension. Sustainability 11, 685 (2019).

49.Van Aelst, K. & Holvoet, N. Climate change adaptation in the Morogoro Region of Tanzania: women's decision-making participation in small-scale farm households. Climate and Development 10, 495–508 (2018).

50.Touch, V., Martin, R. J., Scott, J. F., Cowie, A. & Li Liu, D. Climate change adaptation options in rainfed upland cropping systems in the wet tropics: A case study of smallholder farms in North-West Cambodia. Journal of environmental management 182, 238–246 (2016).

51.Mwase, W., Mtethiwa, A. T. & Makonombera, M. Climate Change adaptation practices for two communities in Southern Malawi. Methodology 4, (2014).

52.Fagariba, C. J., Song, S., Baoro, S. & Gildas, S. K. Climate change adaptation strategies and constraints in Northern Ghana: Evidence of farmers in Sissala West District. Sustainability 10, 1484 (2018).

53.Khanal, U., Wilson, C., Lee, B. L. & Hoang, V.-N. Climate change adaptation strategies and food productivity in Nepal: a counterfactual analysis. Climatic Change 148, 575–590 (2018).

54.Tambo, J. A. & Abdoulaye, T. Climate change and agricultural technology adoption: the case of drought tolerant maize in rural Nigeria. Mitigation and Adaptation Strategies for Global Change 17, 277–292 (2012).

55.Zhai, S. Y., Song, G. X., Qin, Y. C., Ye, X. Y. & Leipnik, M. Climate change and Chinese farmers: Perceptions and determinants of adaptive strategies. Journal of integrative agriculture 17, 949–963 (2018).

56.Alauddin, M. & Sarker, M. A. R. Climate change and farm-level adaptation decisions and strategies in drought-prone and groundwater-depleted areas of Bangladesh: an empirical investigation. Ecological Economics 106, 204–213 (2014).

57.Akinyemi, F. O. Climate change and variability in Semiarid Palapye, Eastern Botswana: An assessment from smallholder farmers' perspective. Weather, Climate, and Society 9, 349–365 (2017).

58.Mutekwa, V. T. Climate change impacts and adaptation in the agricultural sector: The case of smallholder farmers in Zimbabwe. Journal of Sustainable Development in Africa 11, 237–256 (2009).

59.Fagariba, C. J., Song, S. & Baoro, S. K. G. S. Climate change in Upper East Region of Ghana; challenges existing in farming practices and new mitigation policies. Open Agriculture 3, 524–536 (2018).

60.Abraham, F., Bamidele, F. S., Adebola, A. J. & Kobe, I. H. Climate change mitigation activities and determinants in the rural Guinea Savannah of Nigeria. Sustainable Agriculture Research 1, (2012).

61.Tesfaye, W. & Seifu, L. Climate change perception and choice of adaptation strategies. International Journal of Climate Change Strategies and Management (2016).

62.Okoba, B., Dejene, A. A. & Mallo, M. Climate shocks, perceptions and coping options in semi-arid Kenya. in Experiences of climate change adaptation in Africa 167–181 (Springer, 2011).

63.TRAORE, K., SIDIBE, D. & COULIBALY, H. Climate Smart Agriculture as Final Goal: Use of Improved Cereals Varieties in Cinzana, Mali. Journal of Agricultural Studies (2016).

64.Traore, B. et al. Climate variability and change in southern Mali: learning from farmer perceptions and on-farm trials. Experimental Agriculture 51, 615–634 (2015).

65.Bozzola, M., Smale, M. & Di Falco, S. Climate, shocks, weather and maize intensification decisions in rural Kenya. in Agricultural Adaptation to Climate Change in Africa 107–128 (Routledge, 2018).

66.Mubiru, D. N. et al. Climatic trends, risk perceptions and coping strategies of smallholder farmers in rural Uganda. (2015).

67.Tougiani, A., Guero, C. & Rinaudo, T. Community mobilisation for improved livelihoods through tree crop management in Niger. GeoJournal 74, 377 (2009).

68.Kansiime, M. K. Community-based adaptation for improved rural livelihoods: a case in eastern Uganda. Climate and Development 4, 275–287 (2012).

69.Suwanmontri, P., Kamoshita, A., Jongdee, B., Fukai, S. & Kishino, H. Comparative analysis of farmers engaged in participatory research to cope with climate change versus non-participants in Northeast Thailand. Plant Production Science 21, 287–301 (2018).

70.Assoumana, B. T., Ndiaye, M., Puje, G., Diourte, M. & Graiser, T. Comparative assessment of local farmers' perceptions of meteorological events and adaptations strategies: Two Case Studies in Niger Republic. J. Sustain. Dev 9, 118–135 (2016).

71.Assan, E., Suvedi, M., Schmitt Olabisi, L. & Allen, A. Coping with and adapting to climate change: a gender perspective from smallholder farming in Ghana. Environments 5, 86 (2018).

72.Bhatta, G. D. & Aggarwal, P. K. Coping with weather adversity and adaptation to climatic variability: a cross-country study of smallholder farmers in South Asia. Climate and Development 8, 145–157 (2016).

73.Shongwe, P. Cost Benefit Analysis of Climate Change Adaption Strategies on Crop Production Systems: A Case of Mpolonjeni Area Development Programme (ADP) in Swaziland. (2013).

74.Lunduka, R., Fisher, M. & Snapp, S. Could farmer interest in a diversity of seed attributes explain adoption plateaus for modern maize varieties in Malawi? Food Policy 37, 504–510 (2012).

75.Westengen, O. T. & Brysting, A. K. Crop adaptation to climate change in the semi-arid zone in Tanzania: the role of genetic resources and seed systems. Agriculture & Food Security 3, 3 (2014).

76.Moniruzzaman, S. Crop choice as climate change adaptation: Evidence from Bangladesh. Ecological Economics 118, 90–98 (2015).

77.Dalton, T. J., Yesuf, M. & Muhammad, L. Demand for drought tolerance in Africa: selection of drought tolerant maize seed using framed field experiments. (2011).

78.Bernard, M., Hellin, J., Nyikal, R. A. & Mburu, J. G. Determinants for use of certified maize seed and the relative importance of transaction costs. (2010).

79.James, Z. & Julius, M. Determinants of choice of crop variety as climate change adaptation option in arid regions of Zimbabwe. Russian journal of agricultural and socio-economic sciences 15, (2013).

80.Taruvinga, A., Visser, M. & Zhou, L. Determinants of rural farmers' adoption of climate change adaptation strategies: evidence from the Amathole District Municipality, Eastern Cape Province, South Africa. International Journal of Environmental Science and Development 7, 687 (2016).

81.Amare, A. & Simane, B. Determinants of smallholder farmers' decision to adopt adaptation options to climate change and variability in the Muger Sub basin of the Upper Blue Nile basin of Ethiopia. Agriculture & food security 6, 64 (2017).

82.Oo, A. T., Van Huylenbroeck, G. & Speelman, S. Determining factors for the application of climate change adaptation strategies among farmers in Magwe District, dry zone region of Myanmar. International Journal of Climate Change Strategies and Management (2017).

83.Chen, M. et al. Diversification and intensification of agricultural adaptation from global to local scales. PloS one 13, (2018).

84.Houngbo, E. N. Diversité et critères d'adoption des cultivars de maïs (Zea mays L.) dans le village Zounnou, Centre Bénin. Journal of Applied Biosciences 96, 9094–9101 (2015).

85.Khanal, U., Wilson, C., Lee, B. & Hoang, V.-N. Do climate change adaptation practices improve technical efficiency of smallholder farmers? Evidence from Nepal. Climatic change 147, 507–521 (2018).

86.Teklewold, H., Mekonnen, A., Kohlin, G. & Di Falco, S. DOES ADOPTION OF MULTIPLE CLIMATE-SMART PRACTICES IMPROVE FARMERS'CLIMATE RESILIENCE? EMPIRICAL EVIDENCE FROM THE NILE BASIN OF ETHIOPIA. Climate Change Economics 8, 1750001 (2017).

87.Tang, L. & Yu, X. Does the Adoption of Weather Tolerant Variety Contribute to Reduction in Rice Yield Loss? Panel Data Survey from Chinese Rice Farmers. (2016).

88.Murendo, C., Keil, A. & Zeller, M. Drought impacts and related risk management by smallholder farmers in developing countries: evidence from Awash River Basin, Ethiopia. Risk Management 13, 247–263 (2011).

89.Fisher, M. et al. Drought tolerant maize for farmer adaptation to drought in sub-Saharan Africa: Determinants of adoption in eastern and southern Africa. Climatic Change 133, 283–299 (2015).

90.Ward, P. S., Makhija, S. & Spielman, D. J. Drought-tolerant rice, weather index insurance, and comprehensive risk management for smallholders: evidence from a multi-year field experiment in India. Australian Journal of Agricultural and Resource Economics (2019).

91.Ward, P. S., Makhija, S. & Spielman, D. J. Drought-tolerant rice, weather index insurance, and comprehensive risk management for smallholders: evidence from a multi-year field experiment in India. Australian Journal of Agricultural and Resource Economics (2019).

92.Khatri-Chhetri, A., Aryal, J. P., Sapkota, T. B. & Khurana, R. Economic benefits of climate-smart agricultural practices to smallholder farmers in the Indo-Gangetic Plains of India. Current Science 1251–1256 (2016).

93.Shumetie, A. & Yismaw, M. A. Effect of climate variability on crop income and indigenous adaptation strategies of households. International Journal of Climate Change Strategies and Management (2018).

94.Wekesa, B. M., Ayuya, O. I. & Lagat, J. K. Effect of climate-smart agricultural practices on household food security in smallholder production systems: micro-level evidence from Kenya. Agriculture & Food Security 7, 80 (2018).

95.Khanal, U., Adhikari, A. & Wilson, C. Evaluating smallholder farmers' demand for rice variety attributes in Nepal. Journal of Crop Improvement 31, 438–452 (2017).

96.Nyasimi, M., Amwata, D., Hove, L., Kinyangi, J. & Wamukoya, G. Evidence of impact: climate-smart agriculture in Africa. (2014).

97.Williams, N. E. & Carrico, A. Examining adaptations to water stress among farming households in Sri Lanka's dry zone. Ambio 46, 532–542 (2017).

98.Tessema, Y. A., Joerin, J. & Patt, A. Factors affecting smallholder farmers' adaptation to climate change through non-technological adjustments. Environmental development 25, 33–42 (2018).

99.Aryal, J. P., Rahut, D. B., Maharjan, S. & Erenstein, O. Factors affecting the adoption of multiple climate-smart agricultural practices in the Indo-Gangetic Plains of India. in Natural Resources Forum vol. 42 141–158 (Wiley Online Library, 2018).

100.Obayelu, O. A., Adepoju, A. O. & Idowu, T. Factors influencing farmers' choices of adaptation to climate change in Ekiti State, Nigeria. Journal of Agriculture and Environment for International Development (JAEID) 108, 3–16 (2014).

101.Makate, C., Makate, M. & Mango, N. Farm household typology and adoption of climate-smart agriculture practices in smallholder farming systems of southern Africa. African Journal of Science, Technology, Innovation and Development 10, 421–439 (2018).

102.Comoé, H., Finger, R. & Barjolle, D. Farm management decision and response to climate variability and change in Côte d'Ivoire. Mitigation and adaptation strategies for global change 19, 123–142 (2014).

103.Lan, L. et al. Farm-level and community aggregate economic impacts of adopting climate smart agricultural practices in three mega environments. PloS one 13, e0207700–e0207700 (2018).

104.Rogé, P., Friedman, A. R., Astier, M. & Altieri, M. A. Farmer strategies for dealing with climatic variability: a case study from the Mixteca Alta region of Oaxaca, Mexico. Agroecology and Sustainable Food Systems 38, 786–811 (2014).

105.Mubaya, C. P. Farmer strategies towards climate variability and change in Zimbabwe and Zambia. (University of the Free State, 2010).

106.Hadgu, G., Fantaye, K. T., Mamo, G. & Kassa, B. Farmers' climate change adaptation options and their determinants in Tigray Region, Northern Ethiopia. African Journal of Agricultural Research 10, 956–964 (2015).

107.Li, S. et al. Farmers' initiative on adaptation to climate change in the Northern Agro-pastoral Ecotone. International Journal of Disaster Risk Reduction 12, 278–284 (2015).

108.Okonya, J. S., Syndikus, K. & Kroschel, J. Farmers' perception of and coping strategies to climate change: Evidence from six Agro-Ecological zones of Uganda. Journal of Agricultural Science 5, 252 (2013).

109.Toulabi Nejad, M. & Sadeghi, K. Farmers' Strategies in the Face of Droughts and Examination of the Factors Affecting those Strategies: A Case Study of Roshtkhar County. Journal of Rural Research 9, 608–627 (2019).

110.Fosu-Mensah, B. Y., Vlek, P. L. & MacCarthy, D. S. Farmers' perception and adaptation to climate change: a case study of Sekyedumase district in Ghana. Environment, Development and Sustainability 14, 495–505 (2012).

111.Tilahun, U. & Bedemo, A. Farmers' perception and adaptation to climate change: Heckman's two stage sample selection model. Ethiopian Journal of Environmental Studies and Management 7, 832–839 (2014).

112.Kidanu, A., Kibret, K., Hajji, J., Mohammed, M. & Ameha, Y. Farmers' perception towards climate change and their adaptation measures in Dire Dawa Administration, Eastern Ethiopia. Journal of Agricultural Extension and Rural Development 8, 269–283 (2016).

113.Fahad, S. & Wang, J. Farmers' risk perception, vulnerability, and adaptation to climate change in rural Pakistan. Land use policy 79, 301–309 (2018).

114.Jianjun, J., Yiwei, G., Xiaomin, W. & Nam, P. K. Farmers' risk preferences and their climate change adaptation strategies in the Yongqiao District, China. Land Use Policy 47, 365–372 (2015).

115.Yamano, T., Rajendran, S. & Malabayabas, M. L. Farmers' self-perception toward agricultural technology adoption: evidence on adoption of submergence-tolerant rice in Eastern India. Journal of Social and Economic Development 17, 260–274 (2015).

116.Udmale, P., Ichikawa, Y., Manandhar, S., Ishidaira, H. & Kiem, A. S. Farmers' perception of drought impacts, local adaptation and administrative mitigation measures in Maharashtra State, India. International Journal of Disaster Risk Reduction 10, 250–269 (2014).

117.Ngigi, M., Mueller, U. & Birner, R. Gender differences in climate change perceptions and adaptation strategies: an intra-household analysis from rural Kenya. Available at SSRN 2747856 (2016).

118.Adzawla, W. & Kane, A. Gender perspectives of the determinants of climate adaptation: The case of livelihood diversification in Northern Ghana. Review of Agricultural and Applied Economics (RAAE) 21, 113–127 (2018).

119.Yila, J. O. & Resurreccion, B. P. Gender perspectives on agricultural adaptation to climate change in drought-prone Nguru local government area in the semiarid zone of northeastern Nigeria. International Journal of Climate Change Strategies and Management (2014).

120.Murage, A. W., Pittchar, J. O., Midega, C. A. O., Onyango, C. O. & Khan, Z. R. Gender specific perceptions and adoption of the climate-smart push–pull technology in eastern Africa. Crop Protection 76, 83–91 (2015).

121.Diiro, G. et al. Gendered analysis of stakeholder perceptions of climate change, and the barriers to its adaptation in Mopti region in Mali. Telangana, India (2016).

122.Molua, E. L. Gendered response and risk-coping capacity to climate variability for sustained food security in Northern Cameroon. International Journal of Climate Change Strategies and Management (2012).

123.Vom Brocke, K. et al. Helping farmers adapt to climate and cropping system change through increased access to sorghum genetic resources adapted to prevalent sorghum cropping systems in Burkina Faso. Experimental Agriculture 50, 284–305 (2014).

124.Virk, D. S. et al. Highly client-oriented breeding: The impact of two upland rice varieties in eastern India. (2005).

125.de Sousa, K. et al. How climate awareness influences farmers' adaptation decisions in Central America? Journal of rural studies 64, 11–19 (2018).

126.Pérez, C. et al. How resilient are farming households, communities, men and women to a changing climate in Africa? (2014).

127.Makate, C., Wang, R., Makate, M. & Mango, N. Impact of drought tolerant maize adoption on maize productivity, sales and consumption in rural Zimbabwe. Agrekon 56, 67–81 (2017).

128.Morris, M. L. Impacts of international maize breeding research in developing countries, 1966-98. (CIMMYT, 2002).

129.Brüssow, K., Faße, A. & Grote, U. Implications of climate-smart strategy adoption by farm households for food security in Tanzania. Food security 9, 1203–1218 (2017).

130.Handschuch, C. & Wollni, M. Improved production systems for traditional food crops: The case of finger millet in Western Kenya. Food security 8, 783–797 (2016).

131.Peñalba, L. M. & Elazegui, D. D. Improving adaptive capacity of small-scale rice farmers: Comparative analysis of Lao PDR and the Philippines. World Applied Sciences Journal 24, 1211–1220 (2013).

132.Alem, Y., Eggert, H. & Ruhinduka, R. Improving welfare through climate-friendly agriculture: The case of the system of rice intensification. Environmental and resource economics 62, 243–263 (2015).

133.Fukai, S. & Ouk, M. Increased productivity of rainfed lowland rice cropping systems of the Mekong region. Crop and Pasture Science 63, 944–973 (2013).

134.Makate, C., Makate, M., Mango, N. & Siziba, S. Increasing resilience of smallholder farmers to climate change through multiple adoption of proven climate-smart agriculture innovations. Lessons from Southern Africa. Journal of environmental management 231, 858–868 (2019).

135.Makate, C. & Makate, M. Interceding role of institutional extension services on the livelihood impacts of drought tolerant maize technology adoption in Zimbabwe. Technology in Society 56, 126–133 (2019).

136.YU, Q. et al. Interpretation of climate change and agricultural adaptations by local household farmers: A case study at Bin County, Northeast China. Journal of Integrative Agriculture 13, 1599–1608 (2014).

137.Cholo, T. C., Fleskens, L., Sietz, D. & Peerlings, J. Is land fragmentation facilitating or obstructing adoption of climate adaptation measures in Ethiopia? Sustainability 10, 2120 (2018).

138.Vasconcelos, A. C. F. et al. Landraces as an adaptation strategy to climate change for smallholders in Santa Catarina, Southern Brazil. Land Use Policy 34, 250–254 (2013).

139.Kattumuri, R., Ravindranath, D. & Esteves, T. Local adaptation strategies in semi-arid regions: study of two villages in Karnataka, India. Climate and Development 9, 36–49 (2017).

140.Caretta, M. A. & Börjeson, L. Local gender contract and adaptive capacity in smallholder irrigation farming: a case study from the Kenyan drylands. Gender, Place & Culture 22, 644–661 (2015).

141.Peterson, C. A. Local-level appraisal of benefits and barriers affecting adoption of climate-smart agricultural practices: Ghana. (2014).

142.Hellin, J., Bellon, M. R. & Hearne, S. J. Maize landraces and adaptation to climate change in Mexico. Journal of Crop Improvement 28, 484–501 (2014).

143.Balemie, K. Management and uses of farmers’ varieties in southwest Ethiopia: a climate change perspective. (2011).

144.Di Falco, S. & Veronesi, M. Managing environmental risk in presence of climate change: the role of adaptation in the Nile Basin of Ethiopia. Environmental and Resource Economics 57, 553–577 (2014).

145.Muita, R. R., Van Ogtrop, F., Ampt, P. & Vervoort, R. W. Managing the water cycle in Kenyan small-scale maize farming systems: Part 1. Farmer perceptions of drought and climate variability. Wiley Interdisciplinary Reviews: Water 3, 105–125 (2016).

146.Ouédraogo, M. et al. Markets and climate are driving rapid change in farming practices in Savannah West Africa. Regional Environmental Change 17, 437–449 (2017).

147.Gotor, E., Fadda, C. & Trincia, C. Matching Seeds to Needs-female farmers adapt to a changing climate in Ethiopia. (2014).

148.Suarez, P. & Linnerooth-Bayer, J. Micro-insurance for local adaptation. Wiley Interdisciplinary Reviews: Climate Change 1, 271–278 (2010).

149.Snapp, S. et al. Modeling and participatory farmer-led approaches to food security in a changing world: A case study from Malawi. Science et changements planétaires/Sécheresse 24, 350–358 (2013).

150.Cavatassi, R., Lipper, L. & Narloch, U. Modern variety adoption and risk management in drought prone areas: insights from the sorghum farmers of eastern Ethiopia. Agricultural Economics 42, 279–292 (2011).

151.Ficiciyan, A., Loos, J., Sievers-Glotzbach, S. & Tscharntke, T. More than yield: Ecosystem services of traditional versus modern crop varieties revisited. Sustainability 10, 2834 (2018).

152.Yamano, T., Malabayabas, M. L., Habib, M. A. & Das, S. K. Neighbors follow early adopters under stress: panel data analysis of submergence-tolerant rice in northern Bangladesh. Agricultural Economics 49, 313–323 (2018).

153.Duncan, J. M. A., Dash, J. & Tompkins, E. L. Observing adaptive capacity in Indian rice production systems. AIMS Agriculture and Food 2, 165–182 (2017).

154.Vincent, K., Joubert, A., Cull, T., Magrath, J. & Johnston, P. Overcoming the barriers: How to ensure future food production under climate change in Southern Africa. Oxfam Policy and Practice: Agriculture, Food and Land 11, 183–242 (2011).

155.Fatuase, A. & Ajibefun, I. Perception and adaptation to climate change among farmers in selected communities of Ekiti State, Nigeria. Gaziosmanpaşa Üniversitesi Ziraat Fakültesi Dergisi 31, 100–113 (2014).

156.Bedeke, S. B., Vanhove, W., Wordofa, M. G., Natarajan, K. & Van Damme, P. Perception of and response to climate change by maize-dependent smallholders. Climate Research 75, 261–275 (2018).

157.Devkota, N., Phuyal, R. K. & Shrestha, D. L. Perception, determinants and barriers for the adoption of climate change adaptation options among nepalese rice farmers. Agricultural Sciences 9, 272–298 (2018).

158.Ramadas, S., Kumar, A., Singh, S. & Kumar, S. Perception, Yield Sensitivity and Adaptation Strategies to Climate Change: Insights from Wheat Production in India. (2018).

159.Tembo, F. M., Tadesse, T. & Singini, W. Perceptions and choices of adaptation measures for climate change among teff (Eragrostis tef) farmers of Southeast Tigray, Ethiopia. Journal of Agricultural Extension and Rural Development 10, 11–19 (2017).

160.Oselebe, H. O. et al. Perceptions of climate change and variability, impacts and adaptation strategies by rice farmers in south east Nigeria. Our Nature 14, 54–63 (2016).

161.Popoola, O. O., Monde, N. & Yusuf, S. F. G. Perceptions of climate change impacts and adaptation measures used by crop smallholder farmers in Amathole district municipality, Eastern Cape province, South Africa. GeoJournal 83, 1205–1221 (2018).

162.Singh, R. K. et al. Perceptions of climate variability and livelihood adaptations relating to gender and wealth among the Adi community of the Eastern Indian Himalayas. Applied Geography 86, 41–52 (2017).

163.Rioux, J. et al. Planning, implementing and evaluating climate-smart agriculture in smallholder farming systems. Mitigation of Climate Change in Agriculture Series (FAO) eng no. 11 (2016).

164.Ali, A. M. S. Population pressure, environmental constraints and agricultural change in Bangladesh: examples from three agroecosystems. Agriculture, ecosystems & environment 55, 95–109 (1995).

165.Hassen, A., Talore, D. G., Tesfamariam, E. H., Friend, M. A. & Mpanza, T. D. E. Potential use of forage-legume intercropping technologies to adapt to climate-change impacts on mixed crop-livestock systems in Africa: a review. Regional environmental change 17, 1713–1724 (2017).

166.Zhang, L., Hu, J., Li, Y. & Pradhan, N. S. Public-private partnership in enhancing farmers' adaptation to drought: Insights from the Lujiang Flatland in the Nu River (Upper Salween) valley, China. Land use policy 71, 138–145 (2018).

167.Tiffen, M. & Mortimore, M. Questioning desertification in dryland sub–Saharan Africa. in Natural Resources Forum vol. 26 218–233 (Wiley Online Library, 2002).

168.Kabote, S. J. et al. Rain-fed farming system at a crossroads in semi-arid areas of Tanzania: what roles do climate variability and change play? Journal of Environment and Earth Science 4, 85–101 (2014).

169.McKune, S. et al. Reaching the end goal: Do interventions to improve climate information services lead to greater food security? Climate Risk Management 22, 22–41 (2018).

170.Comoé, H. & Siegrist, M. Relevant drivers of farmers' decision behavior regarding their adaptation to climate change: a case study of two regions in Côte d'Ivoire. Mitigation and adaptation strategies for global change 20, 179–199 (2015).

171.Rhodes, E. R., Jalloh, A. & Diouf, A. Review of research and policies for climate change adaptation in the agriculture sector in West Africa. Future agricultures working paper 90, (2014).

172.Kolleh, J. B. Rice farmers' perception of climate change and adaptation strategies in the Ketu North district, Volta region of Ghana. (University of Cape Coast, 2015).

173.Jumare, H., Visser, M. & Brick, K. Risk Preferences and the Poverty Trap: A Look at Technology Uptake amongst Smallholder Farmers in the Matzikama Municipality. (2018).

174.Arodokoun, U. et al. Role of the NTIC in adapting to climate change by cotton growers of Central Benin. African Crop Science Journal 20, 409–423 (2012).

175.Maharjan, S. & Maharjan, K. Roles and contributions of community seed banks in climate adaptation in Nepal. Development in Practice 28, 292–302 (2018).

176.Baudoin, M.-A., Sanchez, A. C. & Fandohan, B. Small scale farmers' vulnerability to climatic changes in southern Benin: the importance of farmers' perceptions of existing institutions. Mitigation and Adaptation Strategies for Global Change 19, 1195–1207 (2014).

177.Wood, S. A., Jina, A. S., Jain, M., Kristjanson, P. & DeFries, R. S. Smallholder farmer cropping decisions related to climate variability across multiple regions. Global Environmental Change 25, 163–172 (2014).

178.Jiri, O., Mafongoya, P. & Chivenge, P. Smallholder farmer perceptions on climate change and variability: A predisposition for their subsequent adaptation strategies. Journal of Earth Science & Climatic Change 6, 1–7 (2015).

179.Gbegbelegbe, S. et al. Smallholder farmers in eastern Africa and climate change: a review of risks and adaptation options with implications for future adaptation programmes. Climate and Development 10, 289–306 (2018).

180.Pauline, N. M., Vogel, C., Grab, S. & Liwenga, E. T. Smallholder farmers in the Great Ruaha River sub-Basin of Tanzania: coping or adapting to rainfall variability? Climate and Development 9, 217–230 (2017).

181.Kihupi, M. L., Mahonge, C. & Chingonikaya, E. E. Smallholder farmers' adaptation strategies to impact of climate change in semi-arid areas of Iringa District Tanzania. (2015).

182.Belay, A., Recha, J. W., Woldeamanuel, T. & Morton, J. F. Smallholder farmers' adaptation to climate change and determinants of their adaptation decisions in the Central Rift Valley of Ethiopia. Agriculture & Food Security 6, 24 (2017).

183.Alemayehu, A. & Bewket, W. Smallholder farmers' coping and adaptation strategies to climate change and variability in the central highlands of Ethiopia. Local Environment 22, 825–839 (2017).

184.Abdoulaye, T., Bamire, S. A., Akinola, A. A. & Etwire, P. M. Smallholder farmers' perceptions and strategies for adaptation to climate change in Brong Ahafo and Upper West Regions of Ghana. (2017).

185.Tambo, J. A. & Abdoulaye, T. Smallholder farmers' perceptions of and adaptations to climate change in the Nigerian savanna. Regional Environmental Change 13, 375–388 (2013).

186.Fisher, M. & Snapp, S. SMALLHOLDER FARMERS'PERCEPTIONS OF DROUGHT RISK AND ADOPTION OF MODERN MAIZE IN SOUTHERN MALAWI. Experimental Agriculture 50, 533–548 (2014).

187.Clay, N. & King, B. Smallholders' uneven capacities to adapt to climate change amid Africa's 'green revolution': Case study of Rwanda's crop intensification program. World development 116, 1–14 (2019).

188.Mwongera, C., Boyard-Micheau, J., Baron, C. & Leclerc, C. Social process of adaptation to environmental changes: How eastern African societies intervene between crops and climate. Weather, Climate, and Society 6, 341–353 (2014).

189.Anyoha, N. O. et al. Socio-economic factors influencing climate change adaptation among crop farmers in Umuahia South Area of Abia State, Nigeria. Net Journal of Agricultural Science 1, 42–47 (2013).

190.Holden, S. T. & Fisher, M. Subsidies promote use of drought tolerant maize varieties despite variable yield performance under smallholder environments in Malawi. Food Security 7, 1225–1238 (2015).

191.Jupiter Ndjeunga, 1* Marou A. Zarafi, 2 Albert Nikiema, 1 PS Traore, 1 Abdou Amani, 2 Sabiou Mahamane, 2 AM Ibro, 1 Souleymane Amadou3 and. Sustainable Land and Water Management Approaches in Sub-Saharan Africa: Farm-level Analysis of Climate Change Mitigation and Adaptation from Sub-Saharan Africa. Climate Change Challenges and Adaptations at Farm-level: Case Studies from Asia and Africa 9, 146 (2015).

192.Mullins, J., Zivin, J. G., Cattaneo, A., Paolantonio, A. & Cavatassi, R. The adoption of climate smart agriculture: the role of information and insurance under climate change. in Climate Smart Agriculture 353–383 (Springer, Cham, 2018).

193.Kuntashula, E., Chabala, L. M., Chibwe, T. K. & Kaluba, P. The effects of household wealth on adoption of agricultural related climate change adaptation strategies in Zambia. Sustainable Agriculture Research 4, (2015).

194.Fisher, M. & Carr, E. R. The influence of gendered roles and responsibilities on the adoption of technologies that mitigate drought risk: The case of drought-tolerant maize seed in eastern Uganda. Global Environmental Change 35, 82–92 (2015).

195.Abid, M., Ngaruiya, G., Scheffran, J. & Zulfiqar, F. The role of social networks in agricultural adaptation to climate change: implications for sustainable agriculture in Pakistan. Climate 5, 85 (2017).

196.Gadédjisso-Tossou, A. Understanding farmers' perceptions of and adaptations to climate change and variability: The case of the Maritime, Plateau and Savannah Regions of Togo. Agricultural Sciences 6, 1441 (2015).

197.Ogunsola, G. O., Olugbire, O. O., Oyekale, A. S. & Aremu, F. J. Understanding perception and adaptation to climate change among cocoa farmers in tropical condition. Ethiopian Journal of Environmental Studies and Management 8, 816–825 (2015).

198.Tiyo, C. E., Orach-Meza, F. L. & Edroma, E. L. Understanding Small-Scale Farmers' Perception and Adaption Strategies to Climate Change Impacts: Evidence from Two Agro-Ecological Zones Bordering National Parks of Uganda. Journal of Agricultural Science 7, 253 (2015).

199.Jain, M., Naeem, S., Orlove, B., Modi, V. & DeFries, R. S. Understanding the causes and consequences of differential decision-making in adaptation research: adapting to a delayed monsoon onset in Gujarat, India. Global Environmental Change 31, 98–109 (2015).

200.Nyasimi, M. et al. Uptake and dissemination pathways for climate-smart agriculture technologies and practices in Lushoto, Tanzania. (2016).

201.Guodaar, L., Beni, A. & Benebere, P. Using a mixed-method approach to explore the spatiality of adaptation practices of tomato farmers to climate variability in the Offinso North District, Ghana. Cogent Social Sciences 3, 1273747 (2017).

202.Thierfelder, C., Matemba-Mutasa, R. & Rusinamhodzi, L. Yield response of maize (Zea mays L.) to conservation agriculture cropping system in Southern Africa. Soil and Tillage Research 146, 230–242 (2015).

Ceres2030
Sustainable Solutions to End Hunger

What are determinants that lead small-scale producers in low- and middle-income countries to adopt climate resilient crops and crop-varieties?

A scoping review protocol

Administrative Information

1. **Open Science Framework registration:** https://osf.io/am3kb/

2. **Authors**:

- Maricelis Acevedo, Cornell University, USA, ma934@cornell.edu
- Jaron Porciello, Cornell University, USA, jat264@cornell.edu
- Kate Ghezzi-Kopel, Cornell University, USA, kwg37@cornell.edu
- Karen Cichy, USDA-ARS, USA, karen.cichy@ars.usda.gov
- Sisi Meng, University of Notre Dame, USA, smeng@nd.edu
- Nkulumo Zinyengere, World Bank, USA, nkulumoz@gmail.com
- Hale Tufan, Cornell University, USA, hat36@cornell.edu
- Kevin Pixley, CGIAR, Mexico, K.PIXLEY@CGIAR.ORG
- Abebe Menkir, IITA, Nigeria, a.menkir@cgiar.org
- Livia Bizikova, International Institute of Sustainable Development, Canada, lbizikova@iisd.ca

3. **Roles and responsibilities:**
   - Content expertise: MA, KC, SM, NZ, HT, KP, AB, LB
   - Systematic review methods and information retrieval: KGK, JP

Introduction/Background

The goal of this article is to review experiences and evidence about how climate-resilient (CR) crops and crop-varieties can be successfully introduced, leading to widespread adoption. We focus on climate change vulnerable countries and countries that have experienced climate-related impacts.

For all countries, but especially those that rely on domestic agriculture production for food security, one of the most critical and proactive measures to cope with food insecurity caused by unpredictable weather patterns is for farmers to adopt climate-resilient crops. In this review, we will first define climate-resiliency traits before exploring the social, technical and regulatory factors that have led to the successful adoption of CR crops and crop-varieties. We will look for primary qualitative and quantitative research that describes where and how CR crops have been introduced and adopted. We will also look at the relationship between experience of severe weather events and the introduction and farmer adoption of new varieties. We will look

at literature from the past thirty years, where the documented impacts of climate change started appearing in the literature.

Research question:

What are determinants that lead small-scale producers in low- and middle-income countries to adopt climate resilient crops and crop-varieties?

Methods

1. **Objectives:** The following key elements used to conceptualize the review questions and/or objectives:

   a. **Setting**
   Low and middle-income countries and a subset of those that are climate vulnerable.
      - We use the World Bank's classification of low- and middle- income countries (as of May 1, 2019).
      - We use the Notre Dame Global Adaptation Initiative's definition of climate vulnerability: "a country's vulnerability to climate change and other global challenges in combination with its readiness to improve resilience." The ND-GAIN index score was used to assess the level of vulnerability, ND Gain, https://gain.nd.edu/our-work/country-index/rankings/).

   b. **Population**

   Our target population is small-scale producers in climate-vulnerable countries, where:

      - Definitions of **"small-scale food producers"** that are found in the literature are mostly based on four criteria: land size, labor input (especially of family members), market orientation and economic size (2). Land size is the most commonly used criterion. The vast majority of definitions of "small-scale food producers" are based on the acreage of the farm and/or a headcount of the livestock raised. Sometimes an arbitrary size is created (commonly 2 hectares or less) but otherwise a relative measure is used, that considers the average size of land-holdings in the country, and a poverty measure (farms that are at 40% or less of the median income). A second important criterion of small-scale producer is the source of the labor used on the farm (whether the labor is provided by the household that runs the farm, or is paid a wage). A third criterion is the extent to which the farm output is sold on a market rather than consumed by the farm household, or bartered with neighbors (some authors cautioned that this is also contextual and many small-scale

producers are engaged in commercial markets). A fourth criterion is economic size (the value of the farm's production). **(FAO, 2017).**

- Climate-vulnerable countries are those considered to be vulnerable to climate change and other global challenges in combination with its readiness to improve resilience.

c. Single intervention
Interventions that lead to small scale producers to adopt crops, varieties, germplasm and planting materials that are climate-resilient.

d. Comparators
No comparators

e. Outcome/s of interest

Primary outcomes:
- Adoption rates of new climate resilient crops or crop-varieties

Secondary outcomes:
- improved food security
- increased income of the small-holders
- diversification of diet
- poverty reduction
- Higher yields

f. Study design/publication type
- Original research and reviews of original research

<u>Definitions:</u>

Climate resiliency: Climate resilience can be generally defined as the capacity for a socio-ecological system to: (1) absorb stresses and maintain function in the face of external stresses imposed upon it by climate change and (2) adapt, reorganize, and evolve into more desirable configurations that improve the sustainability of the system, leaving it better prepared for future climate change impacts. We will search the literature starting in 1990, which the same year that the IPCC released its first report on climate change.
constructed.

Climate change impacts: Impacts: Effects on natural and human systems. In this report, the term impacts is used primarily to refer to the effects on natural and human systems of extreme weather and climate events and of climate change. Impacts generally refer to effects on lives, livelihoods, health, ecosystems, economies, societies, cultures, services, and infrastructure due to the interaction of climate changes or hazardous climate events occurring within a specific

time period and the vulnerability of an exposed society or system. Impacts are also referred to as consequences and outcomes. The impacts of climate change on geophysical systems, including floods, droughts, and sea level rise, are a subset of impacts called physical impacts (IPCC, 2014).

Climate change adaptation: adaptation seeks to lower the risks posed by the consequences of climatic changes. Adaptation measures may be planned in advance or put in place spontaneously in response to a local pressure.

Climate-resilient crops are intended to increase crop yields, and thereby provide a means of adapting to diminishing crop yields in the face of droughts, higher average temperatures, and other climatic conditions (SAAB, A. 2016).

Adoption: Stage in which technology has been selected and is being used by an individual or an organization. Acceptance of a new product or innovation.

Gender: The social relations between men and women, boys and girls, and how this is socially constructed. Gender roles are dynamic and change over time.

Gender based constraints: Refer to restrictions on men's or women's access to resources or opportunities that are based on their gender roles or responsibilities

Eligibility criteria

For an article to be included in this study, it must meet all of the following inclusion criteria:

1. Study focus includes population of small-scale food producers (see definitions)
2. Study is published between 1990- start of search (1990 is when first IPCC inter-governmental panel on climate change met; climate change was first described).
3. Original research (qualitative and quantitative reports) and/or review of existing research including gray literature.
4. Explicit focus or clear relevance on climate change resiliency or climate change adaptation (see definition)
5. Explicit focus on crops, varieties, seed, planting materials or germplasm
6. Study mentions factors for adoption (see definition)
7. Study area or focus includes target populations in lower and middle-income countries (See World bank list)

For an article to be excluded from this scoping review, it must meet one of the following exclusion criteria:

1. Study focus does not include population of small-scale food producers (See definitions)
2. Study is published prior to 1990

3. Not original research or a review of existing research
4. No explicit focus or relevance on climate change resiliency or climate change adaptation (See definitions)
5. Study does not explicitly focus on crops, varieties, seed, planting materials or germplasm
6. Study does not mention factors for adoption (see definitions)
7. Study area or focus does not include target populations in lower and middle-income countries (See World Bank list)

Information sources:

We have conducted a comprehensive search of the following electronic databases and grey literature sources:

- CAB Abstracts (access via Web of Science)
- Web of Science Core Collection (access via Web of Science)
- Scopus (access via Elsevier)
- AgEcon Search
- Africa Theses and Dissertations
- AGRIS (FAO-consolidated search)
- Campbell Collaboration
- Cochrane
- Collaboration for Environmental Evidence (CEE)
- Commonwealth Scientific and Industrial Research Organisation (CSIRO)
- French Agricultural Research Centre for International Development (CIRAD)
- Gardian (searches 15 CGIAR websites)
- International Fund for Agricultural Development (IFAD)
- JPAL/ATAI impact evaluations (IPA)
- Overseas Development Institute (ODI)
- UK Department for International Development (DFID)
- World Bank
- Brazilian Agricultural Research Corporation (EMBRAPA)
- World Health Organization (WHO)
- United Nations Environment Programme (UNEP)
- World Food Programme (WFP)

Search strategy:

A comprehensive search strategy was be developed (KGK) to identify all available research pertaining to facilitators that lead small-scale producers in low- and middle-income countries to adopt climate resilient germplasm. Search terms will include variations of the key concepts in the research question: small-scale producers, germplasm, and climate resiliency or climate

stressors. See Appendix A for a presentation of the search strategy in its entirety such that it may be reproduced in CAB Abstracts (accessed via the Web of Science platform).

Study records

1. Data management:

Searches will be performed across all sources listed in section 5 of this protocol, and search results will be de-duplicated to remove redundant citations identified from multiple sources. Titles, abstracts, and keywords of all citations will be exported as .RIS files. We will use machine learning processes to accelerate the screening process. This includes adding machine-derived metadata to for individual citations, such as identifying populations, geographies, interventions, and outcomes of interest. This will allow for accelerated identification of potential articles for exclusion at the title/abstract screening stage.

We will include all full articles in English, Spanish, French, Portuguese and Chinese.

2. Selection process:

Systematic review software, Covidence, will be used for title and abstract and full-text screening decision-making for this scoping review. Article screening will take place in two phases:

- Title and abstract screening of all de-duplicated citations. Citations will be screened for relevance against the inclusion and exclusion criteria listed in section 4 of this protocol.
- Full-text screening of all articles deemed relevant in the title and abstract screening phase. Citations will be screened for relevance against the inclusion and exclusion criteria listed in section 4 of this protocol. Reasons for exclusion will be documented within Covidence.

In both phases, all citations will be reviewed for relevance by two independent reviewers (MA, KC, SM, NZ, HT, KP, AM, LB, KGK). Each citation that meets all of the inclusion criteria at the title and abstract and full-text screening phases will be included. Each citation that meets one of the exclusion criteria at the title and abstract and full-text screening phases will be excluded. All conflicts will be resolved in Covidence by a third, independent reviewer.

3. Data collection process:

We will develop a data extraction template to document all themes of interest listed in section 9 of this protocol (Data Synthesis/Charting) for each included study. The data extraction template will be tested by the review team before use, and data will be extracted by two independent reviewers. Conflicts will be resolved by consensus. If data

of interest is missing from an included study, we will contact the study authors in effort to obtain it.

Critical appraisal of individual sources of evidence:

The proposed evidence synthesis will be informed by methodological guidelines for scoping reviews (Arksey and O'Malley, 2006; Levac et al 2010), versus standards for preparation of systematic review or meta-analyses. A key difference between scoping reviews and systematic reviews is that the former are generally conducted to provide an overview of the existing evidence regardless of methodological quality or risk of bias (Tricco et al, 2018). Due to the expected heterogeneity in study design of the included studies, we may assess individual sources of evidence for methodological quality or risk of bias. We will not perform a formal critical appraisal of all included studies but rather perform selective critical appraisal of groups of similar study types identified within included studies. Quality of data assessment may include appraisal of study replicability, multi-year, multi-location, size of sample population. The final manuscript will include an explanation of how the appraisal aligns with the review methods.

Data synthesis/charting:

To summarize findings and linking it to the question of interest, we will identify, tag and chart the outcomes by a number of criteria including:

- Determinants of adoption (seed availability, previous experience with biotic or abiotic shock, access to outreach or extension information, income/socio-economic status of farmer or HH, farmer education, access to market, access to market information, access to weather/climate information, access to inputs, access to agriculture/farm infrastructure, sex, age, marital status and ethnicity of farmer, government's policies/program), financial instruments (microcredit, insurance)

- Evidence type (quantitative, qualitative, project report, review, original research, participatory/consultative/survey)
    - for quantitative studies consider nature of data:
        - sample size
        - pre- and post-assessment
        - multilocation
        - multi-year
        - randomized trial
        - meta-analysis

- Country/countries of study (selected from the list of World Bank low- and middle-income countries)

- Particular population represented in the study

- Size of farm
- Farmer sex
- Sex of Head-of-household status
- Marital status
- Socio-economic status (factors to consider: Income, education, occupation)
- Particular crop/variety/germplasm represented in the study
- Farmer network or association "membership"

References

Arksey, H., & O'Malley, L. (2005). Scoping studies: towards a methodological framework. International journal of social research methodology, 8(1), 19-32

Campbell Library. (n.d.). Retrieved March 6, 2019, from https://campbellcollaboration.org/library/campbell-systematic-review-templates.html

Covidence. https://www.covidence.org/

IPCC, 2014: Summary for policymakers. In: Climate Change 2014: Impacts, Adaptation, and Vulnerability. Part A: Global and Sectoral Aspects. Contribution of Working Group II to the Fifth Assessment Report of the Intergovernmental Panel on Climate Change [Field, C.B., V.R. Barros, D.J. Dokken, K.J. Mach, M.D. Mastrandrea, T.E. Bilir, M. Chatterjee, K.L. Ebi, Y .O. Estrada, R.C. Genova, B. Girma, E.S. Kissel, A.N. Levy, S. MacCracken, P .R. Mastrandrea, and L.L. White (eds.)]. Cambridge University Press, Cambridge, United Kingdom and New York, NY , USA, pp. 1-32.

Levac, D., Colquhoun, H., & O'Brien, K. K. (2010). Scoping studies: advancing the methodology.Implementation science, 5(1), 69.

Notre Dame Global Adaptation Index. https://gain.nd.edu/our-work/country-index/rankings/

PRISMA-P-checklist.pdf. (n.d.). Retrieved from http://prisma-statement.org/documents/PRISMA-P-checklist.pdfs

Shamseer, L., Moher, D., Clarke, M., Ghersi, D., Liberati, A., Petticrew, M., … Stewart, L. A. (2015). Preferred reporting items for systematic review and meta-analysis protocols (PRISMA-P) 2015: elaboration and explanation. *BMJ*, *349*, g7647. https://doi.org/10.1136/bmj.g7647

SAAB, A. (2016). Climate-Resilient Crops and International Climate Change Adaptation Law. Leiden Journal of International Law, 29(2), 503-528. doi:10.1017/S0922156516000121

Tricco, A. C., Lillie, E., Zarin, W., O'Brien, K. K., Colquhoun, H., Levac, D., … Straus, S. E. (2018). PRISMA Extension for Scoping Reviews (PRISMA-ScR): Checklist and Explanation. *Annals of Internal Medicine*, *169*(7), 467. https://doi.org/10.7326/M18-0850

Appendix A

Search strategy for CAB Abstracts (Web of Science platform)

Row #	Search string
**1**	TS=("smallhold*" OR "small hold*" OR "small farm*" OR "microfarm*" OR "micro-farm" OR "pastoral" OR "family-run farm*" OR "family-owned farm*" OR "family-managed farm*" OR "agropastoral" OR "agro-pastoral" OR "ejido"OR "campesino")
**2**	TS=(("small-scale" OR "smallscale" OR "low-income" OR "subsistence" OR "semi-subsistence" OR "resource-poor" OR "resource-limited" OR "small-size*" OR "low-income" OR "peasant" OR "district*" OR "village" OR "local" OR "household*") NEAR/3 ("farm*" OR "mixed-farm*" OR "agricultur*" OR "producer*" OR "grower*" OR "agronomy" OR "husbandry"))
**3**	1 OR 2
**4**	DE=("smallholders" OR "small farms" OR "pastoralism" OR "agropastoral systems" OR "silvopastoral systems" OR "community")
**5**	3 OR 4
**6**	TS=("Plant genetic material*" OR "Breeding line*" OR "landrace*" OR "cultivar*" OR "germplasm" OR "accession*" OR "wild relative*" OR "genotype*" OR "crop*" OR "variet*" OR "fruit*" OR "vegetable*" OR "seed" OR "seeds" OR "stakes" OR "stake" OR "vines" OR "vine" OR "corm" OR "corms" OR "planting material*" OR "vegetative clone*" OR "maize" OR "mais" OR "corn" OR "zea mays" OR "legume*" OR "barley" OR "Hordeum vulgare" OR "millet" OR "Panicum miliaceum" OR "Eleusine coracana" OR "Setaria italica" OR "Pennisetum glaucum" OR "oat" OR "oats" OR "avena sativa" OR "rice" OR "palay" OR "oryza sativa" OR "rye" OR "Secale cereale" Or "sorghum" OR "teff" OR "Eragrostis tef" OR "wheat" OR "Triticum aestivum" OR "Triticum turgidum" OR "arrowroot" OR "araro" OR "manranta arundinacea" OR "cassava" OR "tapioca" OR "manioc" OR "kamoteng kahoy" OR "balanghoy" OR "manihot esculenta" OR "Jerusalem artichoke*" OR "sunchoke*" OR "potato*" OR "lambchoke*" OR "Helianthus tuberosus" OR "patatas" OR "Solanum tuberosum" OR "camote" OR "Ipomoea batatas" OR "Taro" OR "gabi" OR "ordinary taro" OR "cocoyam" OR "dalo" OR "talo" OR "arum" OR "dasheen" OR "Colocasia esculenta" OR "Yam" OR "ubi" OR "Dioscorea alata" OR "Yautia" OR "tannia" OR "karlang" OR

	“palawan” OR “bisol” OR “takudo” OR “Xanthosoma sagittifolium” OR “Banana*” OR “bananier” OR “pisang” OR “saging” OR “Musa spp” OR “Breadfruit” OR “rimas” OR “kulo” “Artocarpus altilis” OR “Plantain” OR “saging” OR “cardaba” OR “saba” OR “Sago palm*” OR “sagu” OR “landang” OR “Metroxylon sagus” OR “Sweet palm*” OR “kaong” OR “Arenga pinnata” OR “Chickpea*” OR “Cicer arietinum” OR “Common bean*” OR “Phaseolus vulgaris” OR “Lentil*” OR “Lens culinaris ssp” OR “Pea*” OR “Pisum sativum” OR “Soybean*” OR “Glycine max” OR “Cowpea*”)
**7**	TS=(“climate resilien*” OR “drought toleran*” OR “tolerant of drought” OR “drought resistan*” OR “climate smart” OR “climate mitigat*” OR “heat toleran*” OR “stress toleran*” OR “salinity toleran*” OR “salt toleran*” OR “water efficien*” OR “climate stab*” OR “yield stab*” OR “wide adapt*” OR “frost toleran*” OR “cold toleran*” OR “climate change adaptation” OR “adapting to climate change” OR “adapt to climate change” OR “adaptation to climate change” OR “adaptive capacity” OR “adaptation capacity” OR “water saving” OR “water use efficiency” OR “WUE” OR “adaptive strateg*”)
**8**	TS=(“climate stress*” OR “drought stress*” OR “abiotic stress*” OR “salinity stress*” OR “saline stress*” OR “salt stress*” OR “heat stress*” OR “cold stress*” OR “temperature stress*” OR “moisture stress*” OR “water deficit stress*” OR “climate shock*” OR “water scarc*” OR “water shortage*” OR “water deficien*” OR “dry season*” OR “climatic variability” OR “weather variability” OR “weather stress*” OR “frost damage*” OR “environmental degradation”)
**9**	7 OR 8
**10**	**5 AND 6 AND 9**
**11**	Refined by: PUBLICATION YEARS: 1990-2019

Section 1 of 7

Data extraction Climate Resilient Crops

Title

Year of Pub

Type of publication

Meets ALL inclusion criteria?

1. Yes
2. No
3. Other

Explain other

Short answer text

Country/Countries of study

Data Collection Start Year

Data Collection End Year

Type of study

1. Qualitative
2. Quantitative
3. Mixed-methods
4. Unknown
5. Other

Explain other

Quality of the paper

1. Low Quality

2. High Quality
3. Not Sure
4. Other

Add more detials about paper quality

Data type

- [ ] sample size
- [ ] pre- and post-assessment
- [ ] multilocation
- [ ] multi-year randomized trial
- [ ] meta-analysis
- [ ] survey
- [ ] review/compilation
- [ ] Other

Extra details

Who is represented in the study?

1. Only small-scale farmers

2. Mostly small-holder farmers

3. Small-scale and mid-size farmers

4. Unknown

5. other

Explain other

Farm type (mark all that apply)

- [ ] crops
- [ ] livestocks
- [ ] livestocks + crops,
- [ ] pastoralists
- [ ] subsistence farming
- [ ] business/market driven farm
- [ ] Other...

Explain other

Are the climate resilient crops been adopted for human consumption or as animal feed?

- [ ] Human consumption

- [ ] Animal feed
- [ ] Human consumption and animal feed
- [ ] unknown
- [ ] other

Explain other

Farmer population/ sample sex ratio or numbers (if dissagregated data available)

Sex of Head-of-household status

- [ ] Male
- [ ] Female
- [ ] Mixed
- [ ] Unknown
- [ ] Other...

Explain other

Marital Status

- [ ] Married

- [ ] Unmarried
- [ ] Widowed
- [ ] Unknown
- [ ] Other...

Explain other

Education-level of head of household or survey respondent

- [ ] Primary
- [ ] Secondary
- [ ] College
- [ ] Vocational
- [ ] Training/extension
- [ ] Unknown
- [ ] Other...

Explain other

What is the primary source of income?

- [ ] Crops

- [ ] livestock
- [ ] Off-farm
- [ ] Unknown
- [ ] Other

Explain other

What is the distance to market (for crops and inputs)?

What is the size of the farm?

- [ ] Less than 1 hectares
- [ ] Less than 2 hectares
- [ ] Greater than 2 hectares
- [ ] Unknown
- [ ] Other...

Explain other

Please review the crops/germplasm from the study. (Use the next question to include additional crops and germplasm not listed here).

Please include any additional crops or germplasm addressed in the study not noted in the above.

Are there any key findings related to determinants of adoption (e.g. what factors contribute to adoption)?

- [ ] seed availability
- [ ] previous experience with biotic or abiotic shock
- [ ] access to outreach or extension information
- [ ] income/socio-economic status of farmer or HH
- [ ] farmer education
- [ ] Market access
- [ ] Weather/climate information
- [ ] Access to inputs
- [ ] Access to agriculture/farm infrastructure
- [ ] Government's policies/program
- [ ] Financial instruments (micro-credit, insurance)
- [ ] Other...

Other determinants of adoption

Did you see any evidence of "dis-adoption" of climate-resilient technology? Please describe

Are farmers adopting new crops or new varieties of crops they were already planting?

- New crops
- New varieties of crops they are already planting
- Other

Explain other

Are the climate resilient crops adopted in isolation or as part of other climate resilient technologies?

- Isolation
- Part of other climate resilient technologies/strategies

What are the other climate resilient technologies/strategies?

What is the primary outcome of the study?

- Income was improved
- Yield was improved
- Both income and yield were improved

- Income was not improved
- Yield was not improved
- Neither yield nor income was improved
- The outcome was explicitly environmental
- Other...

Explain other

After section 1 Continue to next section

Section 2 of 7

Social aspects

Does the paper present evidence that social difference (sex, age, marital status, ethinicity etc) positively or negatively impacts adoption of varieties/crops as climate change mitigation strategies?

- Yes
- No
- Unknown
- Other...

Explain other

Does this study have a social network component? Please select all.

- [ ] cooperative
- [ ] association
- [ ] extension
- [ ] political organization
- [ ] faith-based
- [ ] no social network
- [ ] Other

Explain other

Does the farmer have access to extension services and information?

- [ ] Yes
- [ ] No
- [ ] Unknown
- [ ] Other...

Explain other

Which social difference was identified as impacting adoption (s)?

what is the effect of the social difference, if discernible?

- [ ] positive
- [ ] negative
- [ ] unknown
- [ ] Other

Explain other

Please add in other relevant social details

After section 2 Continue to next section

Section 3 of 7

Economic and infrastructure

Does the farmer have access to agricultural inputs?

- yes
- No
- Unknown
- Other...

Explain other

Is any infrastructure in place?

- Transport
- Energy
- Water
- Communications
- Mechanization
- Other...

Explain other

If you have further details about the infrastructure (such as running water, irrigation, etc.) please describe here.

Does the farmer own or rent land?

- ◯ Own
- ◯ Rent
- ◯ Mixed
- ◯ Unknown
- ◯ Other...

Explain other

Does the farmer have access to finance?

- ◯ Community loans
- ◯ Bank loans
- ◯ Remittacnes
- ◯ Other...

Explain other

After section 3 Continue to next section

Section 4 of 7

Environmental aspects

Does the crop/germplasm address any of the following constraints

- [ ] Water efficiency
- [ ] Drought tolerant
- [ ] Salinity tolerant
- [ ] Pest resistant
- [ ] Disease resistant

Please add more details if needed

Method of improved variety/germplasm

- ○ GMO
- ○ Non-GMO
- ○ Other...

Explain other

After section 4 Continue to next section

Section 5 of 7

Other factors

Please list any other factors that are key for the adoption of the crop/germplasm

After section 5 Continue to next section

Section 6 of 7

Cost-modeling information

Can you please indicate the intervention fits into any of the following categories? (Mark all that apply)

Public spending category (if known)

- [ ] ~~Unconditional Cash Transfer: Money given unconditionally to a household or individual.~~
- [ ] ~~Conditional Cash Transfer: Money given conditionally to a household or individual, including food vouchers.~~
- [ ] ~~Food Aid: Physical food items given to a household or individual.~~
- [ ] ~~In-kind: Physical food items given to a household or individual.~~
- [ ] ~~Investment subsidy: Subsidies for investment (that is, credit/loans at a subsidized rate) given to farmers. (Do not include: subsidies for foreign agricultural investment).~~
- [ ] ~~Fertilizer subsidy: Subsidies for fertilizer purchase by farmer. (Do not include: subsidies at parts of the fertilizer value chain other than purchase by farmer).~~
- [ ] ~~Capital endowment: An asset that can be used by a farm/farm household to aid in the farm production process. Assets include machinery, other durable goods, and livestock~~
- [ ]

- [ ] Production subsidy: Subsidies paid to the farmer for the production of agricultural goods.
- [ ] National R&D: Money invested in national agricultural research and development (National Agricultural Research Systems).
- [ ] International R&D: Money invested in international agricultural research and development (e.g., any CGIAR R&D).
- [ ] Extension services: agricultural extension services
- [ ] Storage, on farm: Agricultural product storage interventions that can be used directly by a farm / farm household on their farm.
- [ ] Storage, beyond farm: Agricultural product storage interventions that can be used above the farm level (such as warehouses).
- [ ] Irrigation, on farm: Small-scale irrigation interventions that can be used directly by a singular farm / farm household on their farm.
- [ ] Irrigation, beyond farm: Investment in a public irrigation project.
- [ ] Roads: Investment in a project to build or repair rural roads.
- [ ] OTHER

Explain other

After section 6 Continue to next section

Section 7 of 7

Conclusions

How does study inform policy makers about focal actors?

How does study inform what funders (donors, governments) might want to do?

How does the study inform what actions institutions or other orgs might want to do?

Final thoughts
